# Whole-genome sequencing of genotype VI Newcastle disease viruses from formalin-fixed paraffin-embedded tissues from wild pigeons reveals continuous evolution and previously unrecognized genetic diversity in the U.S.

**DOI:** 10.1186/s12985-017-0914-2

**Published:** 2018-01-12

**Authors:** Ying He, Tonya L. Taylor, Kiril M. Dimitrov, Salman L. Butt, James B. Stanton, Iryna V. Goraichuk, Heather Fenton, Rebecca Poulson, Jian Zhang, Corrie C. Brown, Hon S. Ip, Marcos Isidoro-Ayza, Claudio L. Afonso

**Affiliations:** 1 Southeast Poultry Research Laboratory, Agricultural Research Service, USDA, 934 College Station Rd, Athens, GA 30605 USA; 2 College of Animal Science and Technology, Guangxi University, Nanning, Guangxi China; 3 Department of Pathology, College of Veterinary Medicine, University of Georgia, Athens, GA USA; 4 National Scientific Center Institute of Experimental and Clinical Veterinary Medicine, Kharkiv, Ukraine; 5 Southeastern Cooperative Wildlife Disease Study, Athens, GA USA; 6 Department of Population Health, College of Veterinary Medicine, University of Georgia, Athens, GA USA; 7 National Wildlife Health Center-US Geological Survey, Madison, WI USA; 8 Department of Pathobiological Sciences, School of Veterinary Medicine, University of Wisconsin, Madison, WI USA

**Keywords:** Newcastle disease virus, Formalin-fixed paraffin-embedded, Next-generation sequencing, High-throughput sequencing, NDV, NGS

## Abstract

**Background:**

Newcastle disease viruses (NDV) are highly contagious and cause disease in both wild birds and poultry. A pigeon-adapted variant of genotype VI NDV, often termed pigeon paramyxovirus 1, is commonly isolated from columbids in the United States and worldwide. Complete genomic characterization of these genotype VI viruses circulating in wild columbids in the United States is limited, and due to the genetic variability of the virus, failure of rapid diagnostic detection has been reported. Therefore, in this study, formalin-fixed paraffin-embedded (FFPE) samples were subjected to next-generation sequencing (NGS) to identify and characterize these circulating viruses, providing valuable genetic information. NGS enables multiple samples to be deep-sequenced in parallel. When used on FFPE samples, this methodology allows for retrospective studies of infectious organisms.

**Methods:**

FFPE wild pigeon tissue samples (kidney, liver and spleen) from 10 mortality events in the U.S. between 2010 and 2016 were analyzed using NGS to detect and sequence NDV genomes from randomly amplified total RNA. Results were compared to the previously published immunohistochemistry (IHC) results conducted on the same samples. Additionally, phylogenetic analyses were conducted on the complete and partial fusion gene and complete genome coding sequences.

**Results:**

Twenty-three out of 29 IHC-positive FFPE pigeon samples were identified as positive for NDV by NGS. Positive samples produced an average genome coverage of 99.6% and an average median depth of 199. A previously described sub-genotype (VIa) and a novel sub-genotype (VIn) of NDV were identified as the causative agent of 10 pigeon mortality events in the U.S. from 2010 to 2016. The distribution of these viruses from the North American lineages match the distribution of the Eurasian collared-doves and rock pigeons in the U.S.

**Conclusions:**

This work reports the first successful evolutionary study using deep sequencing of complete NDV genomes from FFPE samples of wild bird origin. There are at least two distinct U.S. lineages of genotype VI NDV maintained in wild pigeons that are continuously evolving independently from each other and have no evident epidemiological connections to viruses circulating abroad. These findings support the hypothesis that columbids are serving as reservoirs of virulent NDV in the U.S.

**Electronic supplementary material:**

The online version of this article (10.1186/s12985-017-0914-2) contains supplementary material, which is available to authorized users.

## Background

Newcastle disease (ND), caused by virulent strains of avian avulavirus 1 (AAvV-1, commonly termed Newcastle disease virus, used hereafter), is a worldwide disease that causes significant economic losses to poultry producers and is exotic to the United States poultry industry [1, 2]. However, genetic variants of Newcastle disease virus (NDV) that belong to genotype VI viruses, termed pigeon paramyxovirus 1 (PPMV-1), are endemic to the U.S. and isolated from wild columbids, including doves and pigeons [3]. Pigeon-origin NDVs are highly contagious and can result in enteric, visceral, and neurological disease in infected birds and has the potential to cause outbreaks in poultry [1].

As with other NDVs, pigeon-origin NDVs are genetically diverse, and their virulence is variable [4]. Although the pigeon-adapted variants are the cause of the third NDV panzootic [4, 5], affecting primarily pigeons and occasionally poultry [1], thorough characterization of the complete genomes of these NDV is limited [6]. Due to the rapid spread of the virus, mass mortalities of specifically Eurasian collared-doves (*Streptopelia decaocto*, ECDO) and rock pigeons (*Columba livia,* ROPI) have occurred within the U.S. [7, 8]. Both species have the ability to interact with naïve species due to the lack of geographical separation, which justifies increased biosecurity within poultry production regions where these columbids abound. For these reasons, it is important to develop a robust and reliable diagnostic assay to provide detailed genomic characterization of NDV circulating in wild columbids in the United States.

Between 2009 and 2016, there were 25 NDV-associated mortality events of pigeons in ten different states of the U.S. [9]. Previously, Isidoro-Ayza, et al. collected 70 birds from 25 events. Using viral isolation, RT-PCR, and immunohistochemistry (IHC), the presence of NDV was confirmed in 14 ECDO and 3 ROPI from 10 of those 25 mortality events [9].

IHC is a common tool in pathogen detection [10–12], it is target-based and can be subjective in some cases. The IHC, using a polyclonal antibody to the nucleoprotein, raised against a synthetic peptide [13], demonstrated widespread NDV antigen in most tissues from most birds. This NDV immunoreactivity, along with the histopathologic changes within these tissues, suggested the lethal pathogenicity of these viruses in these hosts [9]. While IHC can identify only the absence or presence of NDV, molecular methods provide genetic information allowing epidemiological and evolutionary studies to be conducted.

One of the most widely used methods to preserve and archive clinical specimens is formalin fixation and paraffin embedding (FFPE) [14]. Due to the wide use of this technique in pathology studies, significant amount of FFPE samples are stored and available for subsequent analysis. Unlike flash-frozen tissue, nucleic acids are degraded and fragmented through FFPE [15]. Next-generation sequencing (NGS) is a powerful tool used to detect and genotype infectious agents, and a random-priming approach allows unbiased detection of genetic variation [16, 17]. The use of NGS on FFPE samples allows for retrospective studies and for molecular investigations of infectious organisms [18]. While other studies have used NGS on FFPE tissues [19], the use of this approach is limited in studying animal pathogens.

The current aim was to characterize the NDV from the FFPE tissue samples described above [9], and to study the circulation and evolution of endemic pigeon viruses in different regions of the U.S. Here, to improve upon standard NDV genotyping methods, random-priming next-generation sequencing was used for whole-genome (99% coverage) characterization of NDV. The feasibility of NGS to detect, sequence, and identify pathogens in FFPE tissues from wild bird mortality events which have varying degrees of autolysis was demonstrated. This study identified the existence of undescribed genetic diversity of genotype VI NDV in the U.S.

## Methods

### Pigeon FFPE tissue samples

Spleen, kidney and liver FFPE tissue samples from 14 Eurasian collared-doves and 3 rock pigeons, collected from 10 different U.S. mortality events between 2010 and 2016 were used in this study. Samples were collected from the following locations: Plymouth, Massachusetts (MA); Brewster, Texas (TX); Gallatin, Montana (MT); Lea, New Mexico (NM); Hidalgo, TX; Washington, Utah (UT); Dallam, TX; Montgomery, Pennsylvania (PA); and Garden City, Kansas (KS). Tissues were originally fixed in formalin, embedded in paraffin and evaluated by light microscopy and IHC [9] before being analyzed in this study. The same set of organs from one ECDO and one ROPI, negative for NDV and with an unrelated cause of death, were used as negative controls.

### RNA extraction

Five micrometers thick tissue sections were collected in 1.5 ml centrifuge tubes and immediately de-paraffinized by CitriSolv (Thermo Fischer Scientific, USA). Total RNA was extracted using RNeasy FFPE Kit (Qiagen, USA) as per manufacturer’s instructions. Briefly, de-paraffinized tissue was digested by proteinase K, treated with DNase, precipitated by 100% ethanol, adsorbed to an RNeasy MinElute spin column, washed twice and eluted in 50 μl RNase-free water directly from the spin column membrane. Total RNA was quantified using RNA High Sensitivity Assay kits (Thermo Fischer Scientific, USA) on a Qubit® fluorometer 3.0 and fragment size of RNA was determined using RNA 6000 Pico kit on an Agilent Bioanalyzer® (Agilent Technologies, Santa Clara, USA) as per manufacturer’s instructions.

### NGS library preparation, sequencing and genome assembly

Paired-end sequencing was conducted from cDNA libraries prepared from total RNA using KAPA Stranded RNA-Seq kit (KAPA Biosystems, USA) as per manufacturer’s instructions. Briefly, the protocol involved the synthesis of both the first strand and second strand of DNA from total extracted RNA by using random-priming, marking and A-tailing of cDNA fragments with 3′-adenine residues to allow ligation of 3′-thymine-containing adaptors, and random PCR amplification to create the DNA library. The fragment length distribution for each library was assessed using the Agilent High Sensitivity DNA Kit (Agilent Technologies, USA) on the Agilent 2100 Bioanalyzer (Agilent Technologies, USA). The Qubit® fluorometer and the Qubit® dsDNA HS Assay Kit was used to measure the concentration of the dsDNA libraries. All libraries for NGS underwent equimolar dilution and were pooled. The library pool was loaded into the 300 cycle MiSeq Reagent Kit v2 (Illumina, USA) and pair-end sequencing (2 × 150 bp) was performed on the Illumina MiSeq instrument (Illumina, USA). After automated cluster generation in MiSeq, the sequencing reads were processed by bioinformatics. Pre-processing and *de-novo* assembly of the raw sequencing data was completed within the Galaxy platform [20] using a previously described approach [17].

### Phylogenetic analyses

Phylogenetic analyses were performed using MEGA6 software (MEGA, version 6) [21]. Preliminary analysis was performed to infer the evolutionary history of genotype VI NDV. The sequences obtained here along with 82 complete genome concatenated coding sequences (CDS), 305 complete fusion gene sequences, and 1234 sequences of the initial 374 nucleotides (nt) of the fusion gene, which were available in GenBank, were initially analyzed (data not shown). Smaller datasets, including the most closely related viruses (complete genome CDS, *n* = 41, complete fusion gene *n* = 72, and 374 nt partial fusion *n* = 931) (see Additional file 1: Tables S1, S2, and S3), were parsed from the initial datasets and further analyzed. Determination of the best-fit substitution models was performed using MEGA6, and the goodness-of-fit for each model was measured by corrected Akaike information criterion corrected (AICc) and Bayesian information criterion (BIC) [22]. The final trees with 1000 bootstrap replicates were constructed using the maximum-likelihood method based on the General Time Reversible model for the complete genome CDS tree, the Tamura-Nei model for the fusion tree, and Kimura 2-parameter model for the 374 nt partial fusion tree as implemented in MEGA 6. For all analyses, the codon positions included were 1st +, 2nd +, 3rd +, noncoding, and all positions containing gaps and missing data were eliminated.

The estimates of average evolutionary distances were inferred using the complete fusion gene sequences. Analyses were conducted using the maximum composite likelihood model [23]. The rate variation among sites was modeled with a gamma distribution (shape parameter = 1). Previously described criteria based on the phylogenetic topology and evolutionary distances between different taxonomic groups were used to determine genotypes and sub-genotypes [24]. In addition, the USDA validated real-time RT-PCR assay primers and probe [25] used to detect virulent pigeon NDV were aligned with all available sequences obtained from pigeons in the U.S. utilizing ClustalW [26] as implemented in MEGA6.

## Results

### Next-generation sequencing of FFPE tissue samples

Forty-eight FFPE tissue samples (kidney, liver, and spleen) from 17 pigeons, which were collected postmortem in seven states of the U.S., were sequenced using a target-independent NGS approach through random sequencing, and analyzed through the bioinformatics workflow described previously [17]. Twenty-nine of the 48 tissue samples analyzed by NGS were IHC positive [9]. From 17 birds submitted for sequencing, nearly complete genomes were recovered from 12 ECDO and 2 ROPI birds, which represents a total positive characterization rate of 82% (Table 1). Out of 48 tested samples, 23 nearly complete genomes were recovered, and they were all identified as members of genotype VI NDV. The identity among sequences obtained from different tissues of the same bird was 100%. In all sequences, the deduced amino acid cleavage site of the fusion protein was _112_RRKKR↓F_117_, which is specific for virulent viruses. Those positive samples were 87.5% of the total kidneys, 40% of the total spleens, and 18% of the total livers tested. All negative control samples were negative using this NGS approach. The comparison with IHC showed that the 23 samples that were identified as positive for genotype VI by NGS, were also previously identified by Isidoro-Ayza, et al. as positive NDV samples by IHC (Table 1). Interestingly, six samples that were originally examined and classified as IHC positive (21%) were determined to be NGS negative with no NDV detected (see Additional file 2: Table S4). Aligning of the USDA validated real-time RT-PCR assay primers and probe [25] used to detect virulent pigeon NDV to all available sequences obtained from pigeons in the U.S. showed two to four mismatches in each of the oligonucleotides (data not shown).

**Table 1:** Summary of next-generation sequencing data and percent coverage of Newcastle disease virus genomes obtained from formalin-fixed paraffin-embedded tissue samples of wild pigeons collected in the U.S. between 2010 and 2016

SEPRL ID	Tissue	Species	State^a^	Year	NDV reads^e^	% cover.^f^	IHC^g^
**1177-1** ^**b**^	**Kidney**	**ECDO** ^**c**^	**MT**	**2010**	**26,069**	**99.8**	**+**
**1177-2**	**Liver**				**56,910**	**99.8**	**+**
**1177-3**	**Spleen**				**8030**	**99.77**	**+**
1178-2	Liver	ECDO	NM	2013			+
1178-3	Spleen						+
**1179-1**	**Kidney**	**ECDO**	**TX**	**2014**	**1108**	**99.01**	**+**
1179-2	Liver						N
1179-3	Spleen						N
**1180-1**	**Kidney**	**ECDO**	**TX**	**2014**	**14,317**	**99.81**	**+**
**1180-2**	**Liver**				**1761**	**99.64**	**+**
**1180-3**	**Spleen**				**7160**	**99.76**	**+**
**1181-1**	**Kidney**	**ECDO**	**UT**	**2015**	**111,778**	**99.87**	**+**
**1181-2**	**Liver**				**8780**	**99.76**	**+**
**1181-3**	**Spleen**				**32,218**	**99.86**	**+**
**1182-1**	**Kidney**	**ECDO**	**TX**	**2016**	**6387**	**99.84**	**+**
1182-2	Liver						N
**1182-3**	**Spleen**				**32,488**	**99.84**	**+**
1183-1	Kidney	ECDO	TX	2015			+
1183-3	Liver						N
**1184-1**	**Kidney**	**ECDO**	**TX**	**2015**	**2095**	**99.49**	**+**
1184-2	Liver						N
1184-3	Spleen						N
**1185-1**	**Kidney**	**ECDO**	**TX**	**2015**	**2292**	**99.58**	**+**
1185-2	Liver						N
1185-3	Spleen						N
1187-1	Kidney	ROPI^d^	PA	2011			N
1187-2	Liver						N
1187-3	Spleen						N
**1188-1**	**Kidney**	**ROPI**	**MA**	**2014**	**10,533**	**99.84**	**+**
1188-2	Liver						N
**1188-3**	**Spleen**				**1373**	**98.72**	**+**
**1189-1**	**Kidney**	**ROPI**	**PA**	**2012**	**732**	**97.34**	**+**
1189-2	Liver						N
1189-3	Spleen						N
**1191-1**	**Kidney**	**ECDO**	**KS**	**2016**	**37,195**	**99.84**	**+**
1191-2	Liver						+
**1191-3**	**Spleen**				**20,824**	**99.77**	**+**
**1192-1**	**Kidney**	**ECDO**	**KS**	**2016**	**2495**	**99.57**	**+**
1192-2	Liver						N
1192-3	Spleen						+
**1193-1**	**Kidney**	**ECDO**	**KS**	**2016**	**8247**	**99.84**	**+**
1193-2	Liver						N
**1194-1**	**Kidney**	**ECDO**	**KS**	**2016**	**32,269**	**99.83**	**+**
1194-2	Liver						N
1194-3	Spleen						N
**1195-1**	**Kidney**	**ECDO**	**KS**	**2016**	**5481**	**99.82**	**+**
1195-2	Liver						N
1195-3	Spleen						+

^a^Abbreviations of states: *MT* Montana, *PA* Pennsylvania, *TX* Texas, *NM* New Mexico, *UT* Utah, *MA* Massachusetts, *KS* Kansas

^b^Positive samples are in bold font

^c^ECDO (Eurasian collared-dove)

^d^ROPI (rock pigeon)

^e^Numbers of paired reads used to re-call the final NDV consensus for each sequence

^f^Percent coverage - the fraction of the expected full genome length covered by the consensus scaffold

^g^IHC (immunohistochemistry); N (negative); + (positive)

The sequencing of 23 positive NDV samples produced a total of 8,548,074 raw reads, which were reduced to 2,624,877 (30.71%) reads once the host genome and the internal PhiX control were filtered out. The assembly analyses showed that 430,542 reads were mapped to genotype VI NDV. The mean fragment lengths ranged from 121 to 226 nucleotides and the assembled genomes had a percent coverage ranging from 97.34 to 99.86% (Table 1). The mean depth was 9–1498 with 19 out of 23 samples having a mean depth above 20. Detailed information on total and filtered number of reads and fragment length size for each individual sample is provided in Additional file 3, Table S5.

### Phylogenetic analysis

The evolutionary distances between the U.S. pigeon sequences and other genotype VI viruses were estimated. Seventeen sequences obtained in the current study from tissues sampled between 2014 and 2016 from Texas, Utah and Kansas were closely related to each other (99.8% mean identity within the group). These sequences were most closely related (96.8 to 98%) to sub-genotype VIa sequences from Texas and Louisiana from 2004, 2006 and 2010 and single sequences from Rhode Island (RI) (2000), Nevada (2003) and Minnesota (MN) (2007). Three sequences retrieved from birds sampled in Pennsylvania and Massachusetts in 2012 and 2014, respectively (99.8% mean identity between them), were most closely related to sub-genotype VIa sequences recovered from ROPI from Michigan (MI), Maryland (MD) and Pennsylvania in 2013 (99.3–99.4% nucleotide distance) and from pigeons and chickens from Pennsylvania, Minnesota and New Jersey (NJ) between 2004 and 2009 (98.2–98.6% distance). Three more sequences, obtained from a single ECDO sampled in Montana in 2010 and identical to each other, were closely related to sequences from the Northeast U.S. described above and also Connecticut (CT), Maine (ME), New York (NY), and Ohio (OH) during the same period.

**Fig. 1:**
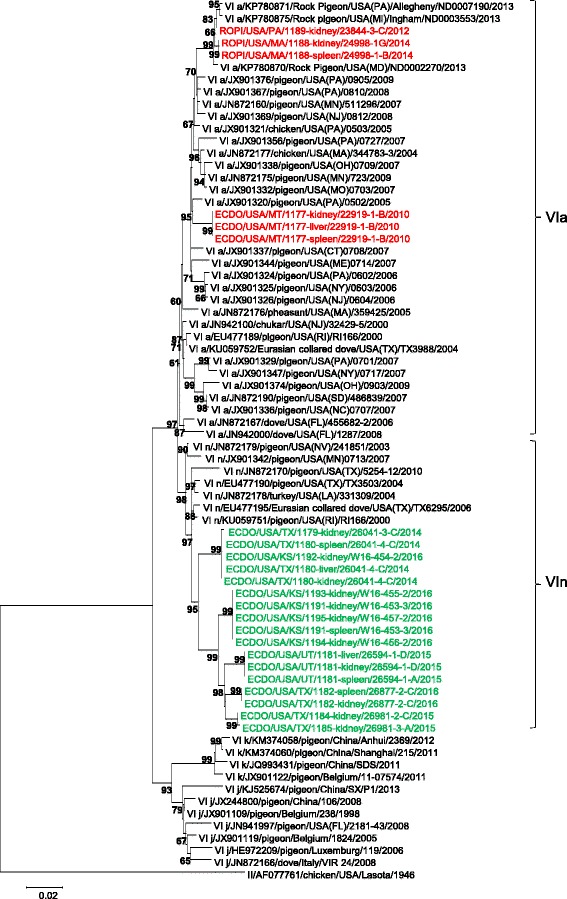
Phylogenetic tree constructed using the complete fusion gene coding sequences of genotype VI Newcastle disease viruses. The evolutionary history was inferred using the Maximum-likelihood method based on the Tamura-Nei model with 1000 bootstrap replicates. The analysis involved 72 complete fusion gene nucleotide sequences of genotype VI, and there were a total of 1662 positions in the final dataset. The NDV sequences obtained from this study are highlighted in either *green* (VIn) or *red* (VIa) colored font, and the distinction between all VIa and VIn viruses is designated by brackets and Roman numerals for the sub-genotype VI designation. The taxa names are labeled by the sub-genotype using Roman numerals, the GenBank identification number, the host organism, the collection country with state designation if from the U.S., the strain designation, and the year of collection. Evolutionary analyses were conducted in MEGA6 [21]

**Fig. 2:**
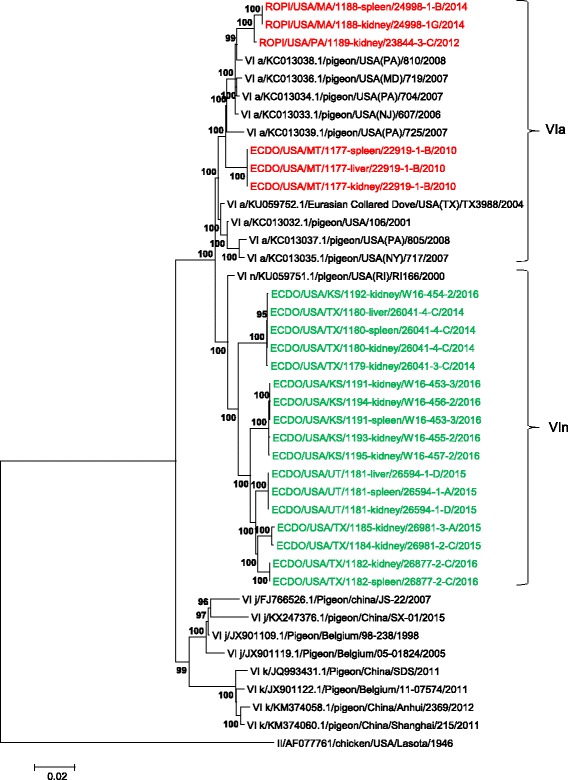
Phylogenetic tree constructed using the complete genome concatenated coding sequences of genotype VI Newcastle disease viruses. The evolutionary history was inferred using the Maximum-likelihood method based on the General Time Reversible model with 1000 bootstrap replicates. The analysis involved 41 complete genome concatenated coding sequences of genotype VI, and there were a total of 13,744 positions in the final dataset. The NDV sequences obtained from this study are highlighted in either *green* (VIn) or *red* (VIa) colored font, and the distinction between all VIa and VIn viruses is designated by brackets and Roman numerals for the sub-genotype VI designation. The taxa names are labeled by the sub-genotype using Roman numerals, the GenBank identification number, the host organism, the collection country with state designation if from the U.S., the strain designation, and the year of collection. Evolutionary analyses were conducted in MEGA6 [21]

In order to confirm the evolutionary relationship between the NDV sequences studied here and viruses isolated in other geographical regions, phylogenetic analyses using the complete genome concatenated CDS, complete fusion gene and 374 nt partial fusion gene sequences were performed. Currently, the accepted method for NDV classification uses the complete coding sequence of the fusion gene [24]. The isolates used in the phylogenetic analysis of the complete coding sequence of the fusion gene expectedly grouped together within currently designated sub-genotype VIa with the viruses that showed highest nucleotide sequence identity to them (Fig. 1). The 17 isolates from Texas, Utah and Kansas, that showed high genetic similarity within the lineage, formed a separate branch with other isolates from Texas, Louisiana, Minnesota, Nevada and Rhode Island that shared high genetic identity with them (Fig. 1). The remaining six sequences studied here clustered together in a separate branch with the sequences retrieved from pigeons and chickens sampled in the Northeast between 2004 and 2009 (Fig. 1). To assess the genetic diversity within sub-genotype VIa, the nucleotide distance between these two groups of sequences was estimated and they were found to be distantly related (4.2% nucleotide distance). In addition, both groups were distantly related (7.21–15.30% and 5.70–14.74%, respectively) to the rest of the sub-genotypes in genotype VI (Table 2 green and red, respectively) supporting the designation of a new sub-genotype, namely VIn, as per the nomenclature criteria for NDV put forth by Diel et al. [24]. The results from the complete genome CDS tree, although less representative due to lack of complete genome sequences in GenBank, show that the phylogenetic relationships identified in the complete fusion gene analyses extend to complete genome level (Fig. 2).

**Table 2:**
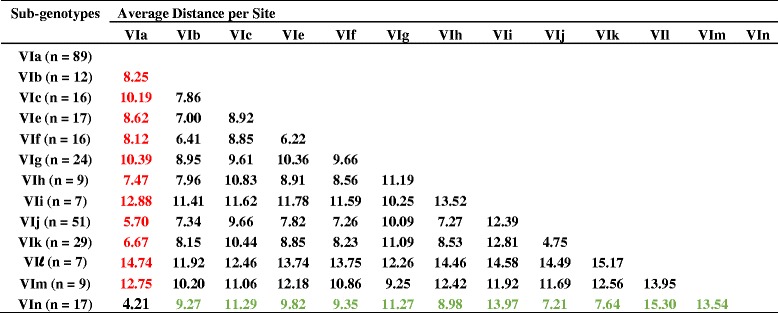
Estimates of evolutionary distances between genotype VI Newcastle disease virus sub-genotypes

The number of base substitutions per site from averaging over all sequence pairs between groups are shown. Analyses were conducted using the Maximum Composite Likelihood model. The rate variation among sites was modeled with a gamma distribution (shape parameter = 1). The analysis involved 307 nucleotide sequences. Codon positions included were 1st + 2nd + 3rd + Noncoding. All positions containing gaps and missing data were eliminated. Evolutionary analyses were conducted in MEGA6

To study the epidemiological relations of the pigeon-origin NDV from the U.S. and the rest of the world, additional phylogenetic analysis was performed utilizing the 374 nt hypervariable region at the beginning of the fusion gene coding sequence (Additional file 4: Figure S1). The analysis was performed on this region due to the abundance of genotype VI 374 nt sequences and the scarcity of complete fusion gene sequences from Europe and China in GenBank. The obtained results suggest that the sub-genotypes VIa and VIn NDV circulating in the U.S. are not directly related to the ones circulating in Europe and Asia. Indeed, viruses from Europe (1987–2005) and the U.S. (isolation from Florida in 2008) grouped into a small branch (Additional file 4: Figure S1, group 1). However, the abundance of sub-genotype VIa viruses from the U.S. (isolated between 2000 and 2014 from North Carolina, South Dakota, PA, RI, TX, MN, NY, NJ, OH, MA, ME, MI, MD, and MT) formed a separate branch in the phylogenetic tree (see Additional file 4: Figure S1, group 2). A third group of sub-genotype VIa sequences was observed, consisting of sequences from Europe (1993–2000), South Africa (2005–2006), Japan (1995–1996) and the U.S. (single isolations from Maryland in 1998 and Florida in 2006) (see Additional file 4: Figure S1, group 3). A fourth branch separated from the latter and contained the isolates identified as novel sub-genotype VIn in the complete fusion gene analysis, along with viruses isolated from Texas, Arizona, Louisiana and Rhode Island between 2000 and 2010 (see Additional file 4, Figure S1, VIn).

## Discussion

The constant evolution of genotype VI NDV has caused failures to detect some viruses in the past [25, 27, 28]. Occasionally, these viruses have been reported to cause outbreaks in poultry, which highlights the necessity to monitor their distribution, circulation and evolution [3, 29–31]. Analysis of the genomic region of the U.S. pigeon NDV used for detection of virulent NDV showed existence of primers/probes mismatches, suggesting that some of these viruses could escape detection by real-time RT-PCR. Due to the need of an unbiased diagnostic approach, we used a previously described target-independent NGS technique [17] that is capable of detecting unknown avian pathogens and applied it on FFPE tissue samples. This is the first report of utilizing random-priming NGS on formalin-fixed, paraffin-embedded tissues from wild birds for evolutionary characterization of Newcastle disease viruses. Newcastle disease viruses of genotype VI, commonly isolated from columbids in the U.S., were identified, and their complete genome sequences were obtained. The performed phylogenetic analysis suggested the existence of a new sub-genotype within genotype VI NDV, thus revealing unknown genetic diversity.

Here, we provide evidence that there are two, independently circulating lineages of genotype VI NDV in the U.S. The phylogenetic analysis showed clear separation of the obtained 23 sequences as they clustered into two independent branches of the phylogenetic tree (Fig. 1). One group had sequences from tissues collected from MT, MA, and PA that clustered with a portion of isolates collected from northeastern states, and the other group had sequences from samples collected from mid-southern states of TX, UT, and KS that clustered with samples from Texas and Louisiana (Fig. 1). The genetic distance between these two groups (4.2% nucleotide distance) suggests a very distant epidemiological link between them. Indeed, such high genetic distance is indicative of at least four decades [32] of independent evolution of these two groups of viruses. Interestingly, the majority of sequences from the mid-south were from ECDO, while the sequences from the northeast were from mixed species including ROPI, unidentified pigeons, doves, chukars, and poultry without prevalence of one species or another, a pattern that could also be noticed in previous studies [7, 33]. The host association of the genotype VI NDV in the southern states with ECDO is puzzling and might reflect the distribution of this species which, although present in many states, predominantly inhabits the southern and southwestern parts of the U.S. with much smaller populations reported in the northeastern states [34, 35]. Because ECDO are an invasive species that first spread throughout Florida in the early 1980s [36, 37], it is possible that this species may have introduced the southern lineage of genotype VI NDV. This is supported by the estimated four decades of evolution described above (4.2% nucleotide distance). However, there is no NDV sequence data from the rest of the world that supports this hypothesis.

The lack of evidence of a direct epidemiological link between U.S. and other genotype VI NDV from other countries and continents suggest the independent maintenance of the U.S. viruses. Utilization of a large number of sequences of the 374 nt region of the fusion gene identified multiple isolations over time of viruses from sub-genotype VIa and sub-genotype VIn across the U.S. (Fig. 1, Additional file 4: Figure S1, group 2, VIn) and only incidental introductions of viruses from abroad. These findings support the hypothesis that columbids are more than likely serving as a reservoir of genotype VI NDV and harboring virulent viruses in the U.S. [1, 3, 38].

The evolutionary distance between the two groups of viruses discussed above, the observed phylogenetic topology, and bootstrap support, indicate the presence of hitherto unidentified genetic diversity among U.S. genotype VI NDV. These two groups fulfill the criteria to be classified as sub-genotypes VIa and VIn [24]. The currently established method to classify NDV based on the full fusion gene analysis provides comprehensive phylogeny (Fig. 1). The complete genome CDS tree supported the results from the full fusion gene analysis and showed bootstrap values of 100 in nearly all nodes, demonstrating the robustness of this analysis (Fig. 2). Furthermore, pigeon viruses from genotype VI have been identified in poultry within the U.S., both in the north and the south, again indicating the potential of these viruses to infect chickens and turkeys (Figs. 1 and 2) and highlighting the importance of keeping the knowledge of their genetic makeup and epidemiology up to date.

The use of FFPE tissues provided several advantages in the current study. This approach allowed complete genome characterization of archival specimens, which were sampled 2 to 7 years ago. The availability to quickly obtain deep, high quality, and detailed molecular characterization of archival samples is of significant value. Previously, the genome of the 1918 pandemic influenza A virus was determined after a 9-year effort by overlapping RT-PCR from postmortem samples; while using NGS, the complete genome was obtained in a single run [39]. Significant numbers of FFPE archival tissues samples dating as far back as the 19th century are stored in tissue banks, research institute repositories and laboratories [39–41]. Sequencing of these samples using our developed approach has the potential to provide a wealth of missing information that will provide insights in genetics, metagenomics and epidemiological knowledge.

Here, the obtained NGS results were compared to the previously performed IHC (see Additional file 2, Table S4). It was confirmed that random-priming NGS of FFPE tissue samples from wild birds can be used as a tool to produce more specific diagnostic information than IHC. While 23 samples were identified as positive for NDV by both NGS and IHC, NGS provided specific genotype and virulence information (see Additional file 2: Table S4). IHC, however, identified only the absence or presence of NDV. Additionally, three birds total, two ECDO and one ROPI, were determined to be negative by NGS (18%) where only one was negative by IHC. Interdependence among the positive detection between the two methods was observed in a high percentage of the samples but not in all of them; nevertheless, NGS provided additional data in all samples. Thus, NGS could be successfully used as a complementary test for precision diagnostics of infectious diseases in FFPE samples.

## Conclusions

This research shows the efficacy of the use of NGS on FFPE samples, as well as its complementary value to IHC, for investigating the circulation and evolution of pigeon-derived genotype VI NDV within the U.S. It demonstrates that there are at least two distinct U.S. genotype VI sub-genotypes maintained in wild pigeons that are continuously evolving independently from each other and independently from the rest of the world.

## Additional files

Additional file 1: Table S1. Dataset used in the complete fusion gene coding sequences phylogenetic analysis. **Table S2.** Dataset used in the complete genome concatenated coding sequences phylogenetic analysis. **Table S3.** Dataset used in the partial fusion gene (374 nucleotides) phylogenetic analysis. (PDF 366 kb)

Additional file 2: Table S4. Comparison of next-generation sequencing results to immunohistochemistry results from 48 Eurasian collared-doves and rock pigeons formalin-fixed paraffin-embedded samples collected in the U.S. between 2010 and 2016. (PDF 208 kb)

Additional file 3: Table S5. The number of raw reads, filtered reads, and mean length of the reads of formalin-fixed paraffin-embedded tissue samples of wild pigeons collected in the U.S. between 2010 and 2016 sequenced by next-generation sequencing (a continuation of Table 1). (PDF 32 kb)

Additional file 4: Figure S1. Phylogenetic analysis using the 374-nucleotide partial fusion gene sequences of genotype VI Newcastle disease viruses. The evolutionary history was inferred using the Maximum-likelihood method based on the Kimura-2 parameter model with 1000 bootstrap replicates [42]. The analysis involved 931 genotype VI partial fusion gene sequences (374 nucleotides). Roman numerals are used for the sub-genotype VI designation, and for a clearer view, the taxa names and bootstrap values are not shown in the radiation tree. The VIa and VIn groups are labeled with the country and years of isolate collection. Evolutionary analyses were conducted in MEGA6 [21]. (PDF 283 kb)

## Abbreviations

AAvV-1: Avian avulavirus 1
AICc: Akaike information criterion corrected
BIC: Bayesian information criterion
CDS: Complete genome concatenated coding sequences
ECDO: Eurasian collared-doves
FFPE: Formalin-fixed paraffin-embedded
IHC: Immunohistochemistry
ND: Newcastle disease
NDV: Newcastle disease viruses
NGS: Next-generation sequencing
nt: nucleotides
PCR: Polymerase Chain Reaction
PPMV-1: Pigeon paramyxovirus 1
ROPI: Rock pigeons
RT-PCR: Reverse Transcription PCR

## References

[1] Miller PJ, Koch G, Swayne DE, Glisson JR, LR MD, Nolan LK, Suarez DL, Nair V (2013). Newcastle disease. Diseases of poultry.

[2] Alexander DJ, Swayne DE, Glisson JR, Jackwood MW, Pearson JE, Reed WM (1998). Newcastle disease virus and other avian Paramyxoviruses. A laboratory manual for the isolation and identification of avian pathogens.

[3] Dimitrov KM, Ramey AM, Qiu X, Bahl J, Afonso CL (2016). Temporal, geographic, and host distribution of avian paramyxovirus 1 (Newcastle disease virus). Infect Genet Evol.

[4] Dortmans JC, Fuller CM, Aldous EW, Rottier PJ, Peeters BP (2010). Two genetically closely related pigeon paramyxovirus type 1 (PPMV-1) variants with identical velogenic fusion protein cleavage sites but with strongly contrasting virulence. Vet Microbiol.

[5] Pearson JE, Senne DA, Alexander DJ, Taylor WD, Peterson LA, Russell PH (1987). Characterization of Newcastle disease virus (avian paramyxovirus-1) isolated from pigeons. Avian Dis.

[6] Brown VR, Bevins SN (2017). A review of virulent Newcastle disease viruses in the United States and the role of wild birds in viral persistence and spread. Vet Res.

[7] Schuler KL, Green DE, Justice-Allen AE, Jaffe R, Cunningham M, Thomas NJ, Spalding MG, Ip HS (2012). Expansion of an exotic species and concomitant disease outbreaks: pigeon paramyxovirus in free-ranging Eurasian collared doves. EcoHealth.

[8] Barton JT, Bickford AA, Cooper GL, Charlton BR, Cardona CJ (1992). Avian paramyxovirus type 1 infections in racing pigeons in California. I. Clinical signs, pathology, and serology. Avian Dis.

[9] Isidoro-Ayza M, Afonso CL, Stanton JB, Knowles S, Ip HS, White CL, Fenton H, Ruder MG, Dolinski AC, Lankton J. Natural infections with pigeon paramyxovirus serotype 1: pathologic changes in Eurasian collared-doves (Streptopelia decaocto) and rock pigeons (Columba livia) in the United States. Vet Pathol. 2017:300985817695782. 10.1177/0300985817695782. 10.1177/0300985817695782 28382855

[10] Lockaby SB, Hoerr FJ, Ellis AC, Yu MS (1993). Immunohistochemical detection of Newcastle disease virus in chickens. Avian Dis.

[11] Gelb J, Fries PA, Peterson FS (1987). Pathogenicity and cross-protection of pigeon paramyxovirus-1 and Newcastle disease virus in young chickens. Avian Dis.

[12] Kommers GD, King DJ, Seal BS, Carmichael KP, Brown CC (2002). Pathogenesis of six pigeon-origin isolates of Newcastle disease virus for domestic chickens. Vet Pathol.

[13] Kommers GD, King DJ, Seal BS, Brown CC (2001). Virulence of pigeon-origin Newcastle disease virus isolates for domestic chickens. Avian Dis.

[14] Berg D, Malinowsky K, Reischauer B, Wolff C, Becker KF (2011). Use of formalin-fixed and paraffin-embedded tissues for diagnosis and therapy in routine clinical settings. Methods Mol Biol.

[15] Kokkat TJ, Patel MS, McGarvey D, LiVolsi VA, Baloch ZW (2013). Archived formalin-fixed paraffin-embedded (FFPE) blocks: a valuable underexploited resource for extraction of DNA, RNA, and protein. Biopreserv Biobank.

[16] Neill JD, Bayles DO, Ridpath JF (2014). Simultaneous rapid sequencing of multiple RNA virus genomes. J Virol Methods.

[17] Dimitrov KM, Sharma P, Volkening JD, Goraichuk IV, Wajid A, Rehmani SF, Basharat A, Shittu I, Joannis TM, Miller PJ, Afonso CL (2017). A robust and cost-effective approach to sequence and analyze complete genomes of small RNA viruses. Virol J.

[18] Klopfleisch R, Weiss AT, Gruber AD (2011). Excavation of a buried treasure--DNA, mRNA, miRNA and protein analysis in formalin fixed, paraffin embedded tissues. Histol Histopathol.

[19] Carrick DM, Mehaffey MG, Sachs MC, Altekruse S, Camalier C, Chuaqui R, Cozen W, Das B, Hernandez BY, Lih CJ (2015). Robustness of next generation sequencing on older formalin-fixed paraffin-embedded tissue. PLoS One.

[20] Afgan E, Baker D, van den Beek M, Blankenberg D, Bouvier D, Cech M, Chilton J, Clements D, Coraor N, Eberhard C (2016). The galaxy platform for accessible, reproducible and collaborative biomedical analyses: 2016 update. Nucleic Acids Res.

[21] Tamura K, Stecher G, Peterson D, Filipski A, Kumar S (2013). MEGA6: molecular evolutionary genetics analysis version 6.0. Mol Biol Evol.

[22] Tamura K, Peterson D, Peterson N, Stecher G, Nei M, Kumar S (2011). MEGA5: molecular evolutionary genetics analysis using maximum likelihood, evolutionary distance, and maximum parsimony methods. Mol Biol Evol.

[23] Tamura K, Nei M, Kumar S (2004). Prospects for inferring very large phylogenies by using the neighbor-joining method. Proc Natl Acad Sci U S A.

[24] Diel DG, da Silva LH, Liu H, Wang Z, Miller PJ, Afonso CL (2012). Genetic diversity of avian paramyxovirus type 1: proposal for a unified nomenclature and classification system of Newcastle disease virus genotypes. Infect Genet Evol.

[25] Kim LM, Afonso CL, Suarez DL (2006). Effect of probe-site mismatches on detection of virulent Newcastle disease viruses using a fusion-gene real-time reverse transcription polymerase chain reaction test. J Vet Diagn Investig.

[26] Thompson JD, Higgins DG, Gibson TJ (1994). CLUSTAL W: improving the sensitivity of progressive multiple sequence alignment through sequence weighting, position-specific gap penalties and weight matrix choice. Nucleic Acids Res.

[27] Kim LM, King DJ, Guzman H, Tesh RB, Travassos da Rosa AP, Bueno R, Dennett JA, Afonso CL (2008). Biological and phylogenetic characterization of pigeon paramyxovirus serotype 1 circulating in wild North American pigeons and doves. J Clin Microbiol.

[28] Sabra M, Dimitrov KM, Goraichuk IV, Wajid A, Sharma P, Williams-Coplin D, Basharat A, Rehmani SF, Muzyka DV, Miller PJ, Afonso CL (2017). Phylogenetic assessment reveals continuous evolution and circulation of pigeon-derived virulent avian avulaviruses 1 in Eastern Europe, Asia, and Africa. BMC Vet Res.

[29] Ayala AJ, Dimitrov KM, Becker CR, Goraichuk IV, Arns CW, Bolotin VI, Ferreira HL, Gerilovych AP, Goujgoulova GV, Martini MC (2016). Presence of vaccine-derived Newcastle disease viruses in wild birds. PLoS One.

[30] Alexander DJ, Parsons G, Marshall R (1984). Infection of fowls with Newcastle disease virus by food contaminated with pigeon faeces. Vet Rec.

[31] Alexander DJ, Wilson GW, Russell PH, Lister SA, Parsons G (1985). Newcastle disease outbreaks in fowl in great Britain during 1984. Vet Rec.

[32] Dimitrov KM, Lee DH, Williams-Coplin D, Olivier TL, Miller PJ, Afonso CL (2016). Newcastle disease viruses causing recent outbreaks worldwide show unexpectedly high genetic similarity to historical virulent isolates from the 1940s. J Clin Microbiol.

[33] Pedersen K, Marks DR, Afonso CL, Stopak SR, Williams-Coplin D, Dimitrov KM, Miller PJ, DeLiberto TJ (2016). Identification of avian paramyxovirus serotype-1 in wild birds in the USA. J Wildl Dis.

[34] Romagosa CM (2012). Eurasian collared-dove (*Streptopelia decaocto*), the birds of North America Ithaca: Cornell lab of ornithology.

[35] Ornithology CLo. eBird: a citizen-based bird observation network in the biological sciences. http://ebird.org/ebird/map/eucdov. Accessed 24 Oct 2017.

[36] Romagosa CM, Labisky RF (2000). Establishment and dispersal of the Eurasian collared-dove in Florida. J Field Ornithol.

[37] Bled F, Royle JA, Cam E (2011). Hierarchical modeling of an invasive spread: the Eurasian collared-dove Streptopelia decaocto in the United States. Ecol Appl.

[38] Rehmani SF, Wajid A, Bibi T, Nazir B, Mukhtar N, Hussain A, Lone NA, Yaqub T, Afonso CL (2015). Presence of virulent Newcastle disease virus in vaccinated chickens in farms in Pakistan. J Clin Microbiol.

[39] Xiao Y, Sheng ZM, Taubenberger JK (2015). Isolating viral and host RNA sequences from archival material and production of cDNA libraries for high-throughput DNA sequencing. Curr Protoc Microbiol.

[40] Fox CH, Johnson FB, Whiting J, Roller PP (1985). Formaldehyde fixation. J Histochem Cytochem.

[41] Tang W, David FB, Wilson MM, Barwick BG, Leyland-Jones BR, Bouzyk MM (2009). DNA extraction from formalin-fixed, paraffin-embedded tissue. Cold Spring Harb Protoc.

[42] Kimura M (1980). A simple method for estimating evolutionary rates of base substitutions through comparative studies of nucleotide sequences. J Mol Evol.

