# Supplementary material for: Whole-genome sequencing of genotype VI Newcastle disease viruses from formalin-fixed paraffin-embedded tissues from wild pigeons reveals continuous evolution and previously unrecognized genetic diversity in the U.S
Source: Virol J. 2018 Jan 12;15:9. doi: 10.1186/s12985-017-0914-2 (PMC5767055; doi:10.1186/s12985-017-0914-2)
Supplement: Supplementary file 1 — Dataset used in the complete fusion gene coding sequences phylogenetic analysis. Table S2. Dataset used in the complete genome concatenated coding sequences phylogenetic analysis. Table S3. Dataset used in the partial fusion gene (374 nucleotides) phylogenetic analysis. (PDF 366 kb) [file 12985_2017_914_MOESM1_ESM.pdf]

Table S1 Dataset used in the complete fusion gene coding sequences phylogenetic analysis.

Genotype	Accession Number	Host	Country	Isolate	Year
II	AF077761	chicken	USA	Lasota	1946
VIa	EU477189	pigeon	USA (RI)	RI166	2000
VIa	JN872160	pigeon	USA (MN)	511296	2007
VIa	JN872167	dove	USA (FL)	455682-2	2006
VIa	JN872175	pigeon	USA (MN)	723	2009
VIa	JN872176	pheasant	USA (MA)	359425	2005
VIa	JN872177	chicken	USA (MA)	344783-3	2004
VIa	JN872190	pigeon	USA (SD)	486839	2007
VIa	JN942000	dove	USA (FL)	1287	2008
VIa	JN942100	chukar	USA (NJ)	32429-5	2000
VIa	JX901320	pigeon	USA (PA)	0502	2005
VIa	JX901321	chicken	USA (PA)	0503	2005
VIa	JX901324	pigeon	USA (PA)	0602	2006
VIa	JX901325	pigeon	USA (NY)	0603	2006
VIa	JX901326	pigeon	USA (NJ)	0604	2006
VIa	JX901329	pigeon	USA (PA)	0701	2007
VIa	JX901332	pigeon	USA (MO)	0703	2007
VIa	JX901336	pigeon	USA (NC)	0707	2007
VIa	JX901337	pigeon	USA (CT)	0708	2007
VIa	JX901338	pigeon	USA (OH)	0709	2007
VIa	JX901344	pigeon	USA (ME)	0714	2007
VIa	JX901347	pigeon	USA (NY)	0717	2007
VIa	JX901356	pigeon	USA (PA)	0727	2007
VIa	JX901367	pigeon	USA (PA)	0810	2008
VIa	JX901369	pigeon	USA (NJ)	0812	2008
VIa	JX901374	pigeon	USA (OH)	0903	2009
VIa	JX901376	pigeon	USA(PA)	0905	2009
VIa	KP780870	ROPI	USA(MD)	ND0002270	2013
VIa	KP780871	ROPI	USA(PA)	ND0007190	2013
VIa	KP780875	ROPI	USA(MI)	ND0003553	2013
VIa	KU059752	ECDO	USA(TX)	TX3988	2004
VIa	24998-1G	ROPI	USA (MA)	1188-1	2014
VIa	22919-1-B	ECDO	USA (MT)	1177-1	2010
VIa	22919-1-B	ECDO	USA (MT)	1177-2	2010
VIa	22919-1-B	ECDO	USA (MT)	1177-3	2010
VIa	24998-1-B	ROPI	USA (MA)	1188-3	2014
VIa	23844-3-C	ROPI	USA (PA)	1189-1	2012
VIj	HE972209	pigeon	Germany	119	2006
VIj	JN872166	dove	Italy	VIR_24	2008

VIj	JN941997	pigeon	USA(FL)	2181-43	2008
VIj	JX244800	pigeon	China	106	2008
VIj	JX901109	pigeon	Belgium	238	1998
VIj	JX901119	pigeon	Belgium	1824	2005
VIj	KJ525674	pigeon	China	SXP1	2013
VIk	JQ993431	pigeon	China	SDS	2011
VIk	JX901122	pigeon	Belgium	11-07574	2011
VIk	KM374058	pigeon	China	2369	2012
VIk	KM374060	pigeon	China	215	2011
VIn	EU477190	pigeon	USA (TX)	TX3503	2004
VIn	EU477195	ECDO	USA (TX)	TX6295	2006
VIn	JN872170	pigeon	USA (TX)	5254-12	2010
VIn	JN872178	turkey	USA (LA)	331309	2004
VIn	JN872179	pigeon	USA (NV)	241851	2003
VIn	JX901342	pigeon	USA (MN)	713	2007
VIn	KU059751	pigeon	USA(RI)	RI166	2000
VIn	26041-3-C	ECDO	USA (TX)	1179-1	2014
VIn	26041-4-C	ECDO	USA (TX)	1180-1	2014
VIn	26041-4-C	ECDO	USA (TX)	1180-2	2014
VIn	26594-1-A	ECDO	USA (UT)	1181-3	2015
VIn	26877-2-C	ECDO	USA (TX)	1182-3	2016
VIn	26981-2-C	ECDO	USA (TX)	1184-1	2015
VIn	26981-3-A	ECDO	USA (TX)	1185-K	2015
VIn	W16-453-3	ECDO	USA (KS)	1191-3	2016
VIn	W16-454-2	ECDO	USA (KS)	1192-1	2016
VIn	W16-456-2	ECDO	USA (KS)	1194-1	2016
VIn	W16-457-2	ECDO	USA (KS)	1195-1	2016
VIn	26041-4-C	ECDO	USA (TX)	1180-3	2014
VIn	W16-455-2	ECDO	USA (KS)	1193-1	2016
VIn	W16-453-3	ECDO	USA (KS)	1191-1	2016
VIn	26877-2-C	ECDO	USA (TX)	1182-1	2016
VIn	26594-1-D	ECDO	USA (UT)	1181-2	2015
VIn	26594-1-D	ECDO	USA (UT)	1181-1	2015

Table S2 Dataset used in the complete genome concatenated coding sequences phylogenetic analysis.

Genotype	Accession Number	Host	Country	Isolate	Year
II	AF077761	chicken	USA	Lasota	1946
VIa	KC013032.1	pigeon	USA	106	2001
VIa	KC013033.1	pigeon	USA(NJ)	607	2006
VIa	KC013034.1	pigeon	USA(PA)	704	2007
VIa	KC013035.1	pigeon	USA(NY)	717	2007
VIa	KC013036.1	pigeon	USA(MD)	719	2007
VIa	KC013039.1	pigeon	USA(PA)	725	2007
VIa	KC013037.1	pigeon	USA(PA)	805	2008
VIa	KC013038.1	pigeon	USA(PA)	810	2008
VIa	KU059752.1	ECDO	USA(TX)	TX3988	2004
VIa	24998-1G	ROPI	USA (MA)	1188-1	2014
VIa	22919-1-B	ECDO	USA (MT)	1177-1	2010
VIa	22919-1-B	ECDO	USA (MT)	1177-2	2010
VIa	22919-1-B	ECDO	USA (MT)	1177-3	2010
VIa	24998-1-B	ROPI	USA (MA)	1188-3	2014
VIa	23844-3-C	ROPI	USA (PA)	1189-1	2012
VIj	JX901119.1	pigeon	Belgium	05-01824	2005
VIj	JX901109.1	pigeon	Belgium	98-238	1998
VIj	FJ766526.1	pigeon	china	JS-22	2007
VIj	KX247376.1	pigeon	China	SX-01	2015
VIk	JX901122.1	pigeon	Belgium	11-07574	2011
VIk	KM374058.1	pigeon	China	2369	2012
VIk	JQ993431.1	pigeon	China	SDS	2011
VIk	KM374060.1	pigeon	China	215	2011
VIn	KU059751.1	pigeon	USA(RI)	RI166	2000
VIn	26041-3-C	ECDO	USA (TX)	1179-1	2014
VIn	26041-4-C	ECDO	USA (TX)	1180-1	2014
VIn	26041-4-C	ECDO	USA (TX)	1180-2	2014
VIn	26594-1-A	ECDO	USA (UT)	1181-3	2015
VIn	26877-2-C	ECDO	USA (TX)	1182-3	2016
VIn	26981-2-C	ECDO	USA (TX)	1184-1	2015
VIn	26981-3-A	ECDO	USA (TX)	1185-K	2015
VIn	W16-453-3	ECDO	USA (KS)	1191-3	2016

VIn	W16-454-2	ECDO	USA (KS)	1192-1	2016
VIn	W16-456-2	ECDO	USA (KS)	1194-1	2016
VIn	W16-457-2	ECDO	USA (KS)	1195-1	2016
VIn	26041-4-C	ECDO	USA (TX)	1180-3	2014
VIn	W16-455-2	ECDO	USA (KS)	1193-1	2016
VIn	W16-453-3	ECDO	USA (KS)	1191-1	2016
VIn	26877-2-C	ECDO	USA (TX)	1182-1	2016
VIn	26594-1-D	ECDO	USA (UT)	1181-2	2015
VIn	26594-1-D	ECDO	USA (UT)	1181-1	2015

Table S3 Dataset used in the partial fusion gene (374 nucleotides) phylogenetic analysis.

Accession Number	Host	Country	Year
EU477196.1	ECDO	USA (TX)	2007
EU477189	pigeon	USA (RI)	2000
JN872178.1	pigeon	USA (LA)	2004
EU477197.1	ECDO	USA (TX)	2007
EU477193.1	ECDO	USA (TX)	2003
EU477191.1	ECDO	USA (TX)	2004
EU477190.1	pigeon	USA (TX)	2004
EU477195.2	ECDO	USA (TX)	2006
EU477192.1	ECDO	USA (TX)	2005
EU477188.1	dove	USA (TX)	2004
JN872170.1	pigeon	USA (TX)	2010
KJ473708.1	ECDO	USA (AZ)	2009
AY471768.1	pigeon	Ireland	1996
JN872183.1	pigeon	USA (MD)	1998
JN872179.1	pigeon	USA (NV)	2003
JN872167.1	dove	USA (FL)	2006
GQ429292.1	pigeon	Netherlands	1996
AY471767.1	pigeon	Germany	1994
AY175753.1	pigeon	Germany	1994
AB070419.1	pigeon	Japan	1995
JX901342.1	pigeon	USA (MN)	2007
JX901317.1	pigeon	USA (PA)	2002
AY471764.1	pigeon	UK	1994
AY471762.1	pigeon	Germany	1994
AY471761.1	pigeon	Denmark	1993
AY150140.1	pigeon	Germany	1994
AY150133.1	pigeon	Germany	1993
AY150132.1	pigeon	Spain	1992
AB070422.1	pigeon	Japan	1996
KU059752.1	ECDO	USA (TX)	2004
JX901369.1	pigeon	USA (NJ)	2008
JX901367.1	pigeon	USA (PA)	2008
JX901360.1	pigeon	USA (PA)	2008
JX901326.1	pigeon	USA (NJ)	2006
JX901325.1	pigeon	USA (NY)	2006
JX901324.1	pigeon	USA (PA)	2006
JX901321.1	chicken	USA (PA)	2005
JX901320.1	pigeon	USA (PA)	2005
JX901314.1	dove	USA (PA)	2001
JX901313.1	pigeon	USA (PA)	2001

KC013038.1	pigeon	USA (PA)	2008
KC013032.1	pigeon	USA (PA)	2001
JN942000.1	dove	USA (FL)	2008
JN872176.1	pheasant	USA (MA)	2005
EU477189.1	pigeon	USA (RI)	2000
AY471759.1	pigeon	Austria	1995
AY471758.1	pigeon	UK	1990
AY150141.1	chicken	Germany	1994
AY150139.1	pigeon	Hungary	1994
JX901376.1	pigeon	USA (PA)	2009
JX901365.1	pigeon	USA (PA)	2008
JX901351.1	pigeon	USA (NJ)	2007
JX901340.1	pigeon	USA (NJ)	2007
JX901339.1	pigeon	USA (NJ)	2007
JX901337.1	pigeon	USA (CT)	2007
JX901334.1	pigeon	USA (PA)	2007
JX901327.1	pigeon	USA (PA)	2006
JX901323.1	pigeon	USA (PA)	2006
JX901319.1	pigeon	USA (PA)	2005
JX901305.1	pigeon	USA (PA)	2003
JX901304.1	pigeon	USA (PA)	2003
JN942100.1	Chukar	USA (NJ)	2000
JN872177.1	chicken	USA (MA)	2004
JN872174.1	Environment	USA (NJ)	2010
JN872160.1	pigeon	USA (MN)	2007
AY471765.1	pigeon	France	1998
AY471760.1	pigeon	Austria	1998
AY150115.1	pigeon	Hungary	1987
JX901372.1	Broiler	USA (PA)	2009
JX901336.1	pigeon	USA (NC)	2007
JN941998.1	pigeon	USA (PA)	2008
JN872190.1	pigeon	USA (SD)	2007
KJ736742.1	pigeon	Germany	1998
JX901375.1	pigeon	USA (PA)	2009
JX901373.1	pigeon	USA (PA)	2009
JX901368.1	pigeon	USA (PA)	2008
JX901355.1	pigeon	USA (PA)	2007
JX901353.1	pigeon	USA (PA)	2007
JX901350.1	pigeon	USA (PA)	2007
JX901344.1	pigeon	USA (ME)	2007
JX901333.1	pigeon	USA (PA)	2007
JX901330.1	pigeon	USA (PA)	2007
JX901329.1	pigeon	USA (PA)	2007

JX901328.1	pigeon	USA (NJ)	2006
KC013034.1	pigeon	USA (PA)	2007
KC013033.1	pigeon	USA (NJ)	2006
JN941999.1	pigeon	USA (MN)	2008
AY288995.1	dove	Italy	2000
AY471771.1	pigeon	AUSAtria	1997
AY562989.1	dove	Italy	2000
AY150149.1	pigeon	Hungary	1998
AY175759.1	dove	Italy	2000
AB070434.1	pigeon	Japan	2000
KP780876.1	pigeon	USA (MI)	2013
KP780875.1	pigeon	USA (MI)	2013
KP780874.1	pigeon	USA (PA)	2013
KP780873.1	pigeon	USA (PA)	2013
KP780872.1	pigeon	USA (PA)	2013
KP780871.1	pigeon	USA (PA)	2013
KP780870.1	pigeon	USA (MD)	2013
KJ473709.1	ECDO	USA (MT)	2009
JX901366.1	pigeon	USA (PA)	2008
JX901364.1	pigeon	USA (PA)	2008
JX901363.1	pigeon	USA (PA)	2008
JX901352.1	pigeon	USA (PA)	2007
JX901349.1	pigeon	USA (MD)	2007
JX901346.1	pigeon	USA (PA)	2007
JX901332.1	pigeon	USA (MO)	2007
KC013036.1	pigeon	USA (MD)	2007
JN941996.1	pigeon	USA (PA)	2008
JN872175.1	pigeon	USA (MN)	2009
GQ429293.1	dove	Italy	2000
DQ904250.1	pigeon	Quebec	2006
AY471766.1	pigeon	UK	1998
KJ473710.1	ECDO	USA (MT)	2010
JX901377.1	pigeon	USA (PA)	2009
JX901359.1	pigeon	USA (PA)	2008
JX901354.1	pigeon	USA (PA)	2007
KC013039.1	pigeon	USA (PA)	2007
JN872172.1	Raptor	USA (MN)	2009
AY471769.1	pigeon	Italy	1999
AY150153.1	pigeon	Hungary	1999
AY150130.1	pigeon	Hungary	1992
AY175752.1	cockatoo	Germany	1995
JX901374.1	pigeon	USA (OH)	2009
JX901361.1	pigeon	USA (PA)	2008

JX901348.1	pigeon	USA (PA)	2007
JX901347.1	pigeon	USA (NY)	2007
JX901338.1	pigeon	USA (OH)	2007
KC013035.1	pigeon	USA (NY)	2007
AY471777.1	pigeon	Portugal	1999
AY445669.1	chicken	South Africa	2002
JX901357.1	pigeon	USA (PA)	2007
DQ289796.1	pigeon	Turkey	2005
AY471773.1	budgerigar	Turkey	1995
JX901362.1	pigeon	USA (PA)	2008
JX901358.1	pigeon	USA (PA)	2008
JX901335.1	pigeon	USA (PA)	2007
KC013037.1	pigeon	USA (PA)	2008
EF030964.1	pigeon	South Africa	2006
EF030956.1	dove	South Africa	2005
EF030955.1	dove	South Africa	2005
EF030950.1	pigeon	South Africa	2006
AY471775.1	pigeon	Portugal	1999
AY150164.1	pigeon	Hungary	2001
AY175776.1	chicken	Turkey	1995
JX901356.1	pigeon	USA (PA)	2007
EF030957.1	dove	South Africa	2006
EF030954.1	dove	South Africa	2005
AY471776.1	pigeon	Portugal	1998
KU527559.1	pigeon	China	2015
EF030962.1	pigeon	South Africa	2004
EF030961.1	dove	South Africa	2006
EF030960.1	dove	South Africa	2006
EF030958.1	dove	South Africa	2006
AY471774.1	pigeon	Portugal	2001
AY471721.1	pigeon	UAE	2000
KJ782376.1	pigeon	China	2011
KJ607166.1	pigeon	China	2013
KC915216.1	pigeon	Macedonia	2012
KC915205.1	pigeon	Macedonia	2007
JX244798.1	pigeon	China	2008
JX901124.1	pigeon	Belgium	2011
JX901110.1	pigeon	Belgium	1998
JX901109.1	pigeon	Belgium	1998
JX901111.1	pigeon	Belgium	1998
JF823994.1	pigeon	Kemerovo	2005
JN986839.1	pigeon	Ireland	2004
EF026583.2	pigeon	Belgium	1998

EF026579.2	pigeon	Belgium	1998
EF030963.1	pigeon	South Africa	2005
EF026582.1	pigeon	Belgium	1998
EF026581.1	pigeon	Belgium	1998
EF026580.1	pigeon	Belgium	1998
AY288996.1	pigeon	Italy	2000
AY471778.1	pigeon	Austria	1997
AY471737.1	pigeon	Ireland	2002
AY471732.1	pigeon	Ireland	2001
AY471725.1	pigeon	UK	2002
AY471723.1	pigeon	UK	2002
AY175770.1	pigeon	UK	1999
AJ306305.1	pigeon	France	2001
KP861633.1	pigeon	China	2012
KF828884.1	pigeon	China	2013
JX901122.1	pigeon	Belgium	2011
JX486552.1	pigeon	China	2011
JN872162.1	Rosella	Belgium	2008
HM776583.1	pigeon	China	2008
HM063425.1	pigeon	China	2010
AY471754.1	pigeon	UK	2002
AY471753.1	pigeon	UK	2001
AY471747.1	pigeon	Belgium	1998
AY471746.1	pigeon	UK	2000
AY471745.1	pigeon	UAE	2000
AY471741.1	chicken	Italy	2000
AY471735.1	pigeon	Canada	1991
AY471730.1	pigeon	UK	2002
AY471728.1	pigeon	UAE	2000
AY471726.1	pigeon	UK	2000
AY471724.1	pigeon	Ireland	2000
AY150155.1	pigeon	Hungary	2000
AY150154.1	pigeon	Germany	2000
AY175756.1	pigeon	Ireland	2000
AY175754.1	pigeon	Germany	1999
AJ306304.1	pigeon	France	2001
KY569335.1	pigeon	Malaysia	2011
KX710210.1	pigeon	India	2015
KU522142.1	pigeon	Egypt	2014
KJ808819.1	pigeon	China	2013
KJ607165.1	pigeon	China	2014
HG326603.1	pigeon	Nigeria	2007
KC915215.1	pigeon	Macedonia	2011

KC915214.1	pigeon	Macedonia	2011
KC915208.1	pigeon	Macedonia	2008
KC915206.1	pigeon	Macedonia	2007
JX244800.1	pigeon	China	2008
JX244799.1	pigeon	China	2008
KC143159.1	pigeon	China	2005
JX901123.1	pigeon	Belgium	2011
JX901118.1	pigeon	Belgium	2003
JQ993431.1	pigeon	China	2011
JQ039391.1	pigeon	Nigeria	2007
JQ039388.1	pigeon	Nigeria	2008
JQ039387.1	pigeon	Nigeria	2008
HM063423.1	White-breasted water hen	China	2005
EF364037.1	pigeon	Slovakia	2005
DQ439884.1	pigeon	China	5
AY471785.1	Kestrel	UAE	1999
AY471783.1	Kestrel	UAE	1998
AY471782.1	pigeon	UAE	1997
AY471781.1	pigeon	UAE	1996
AY471780.1	pigeon	UAE	1999
AY471752.1	pigeon	UK	2001
AY471744.1	pigeon	Ireland	2000
AY471742.1	dove	Italy	2001
AY471736.1	pigeon	UK	2001
AY471727.1	pigeon	UK	2002
AY471722.1	pigeon	Ireland	2000
AY150099.1	pigeon	Hungary	1983
AY175771.1	pigeon	UK	1999
AY175751.1	pigeon	UAE	1996
KX710209.1	pigeon	India	2015
KU377530.1	turtledove	Italy	2004
KU527560.1	pigeon	China	2015
KM374061.1	pigeon	Zhejiang	2012
KJ808820.1	pigeon	China	2012
HG326602.1	pigeon	Nigeria	2007
HG326601.1	pigeon	Nigeria	2007
KC915212.1	pigeon	Macedonia	2010
JX244805.1	pigeon	China	2008
JX244801.1	pigeon	China	2008
JX244794.1	pigeon	China	2008
KC143164.1	pigeon	China	2011
JX901119.1	pigeon	Belgium	2005
JQ979176.1	pigeon	China	2011

EF030953.1	dove	South Africa	2005
DQ202263.1	grouse	Russia	2004
AY734536.1	pigeon	Argentina	1997
AY471779.1	pigeon	UAE	1998
AY471756.1	pigeon	UK	2002
AY471751.1	pigeon	UK	2002
AY471743.1	pigeon	Ireland	2001
AY190518.1	pigeon	Hungary	2002
AY150159.1	pigeon	Sweden	2000
AY150157.1	pigeon	Sweden	2000
AY150092.1	pigeon	Iraq	1978
AF456439.1	Goose	China	1998
KU377529.1	turtledove	Italy	2002
KX761865.1	pigeon	China	2011
KR014815.1	pigeon	China	2014
KT381606.1	pigeon	Guangdong	2014
KT163263.1	pigeon	China	2013
KT163262.1	pigeon	China	2013
KT163261.1	pigeon	China	2012
KM374060.1	pigeon	China	2011
KM374058.1	pigeon	Anhui	2012
KJ525672.1	pigeon	China	2010
KJ607163.1	pigeon	China	2004
KJ600779.1	pigeon	China	2013
KJ600778.1	pigeon	China	2013
KC915213.1	pigeon	Macedonia	2011
JX244803.1	pigeon	China	2008
JX901121.1	pigeon	Belgium	2007
JX486554.1	pigeon	China	2011
JX486551.1	pigeon	China	2005
JN941995.1	pigeon	USA (NY)	2008
JN942095.1	pigeon	USA (OK)	1997
JN872188.1	pigeon	USA (MN)	1989
JN872182.1	pigeon	USA (NY)	1998
JN872180.1	pigeon	USA (TX)	2002
JF713701.1	pigeon	China	2010
HM625835.1	duck	China	2008
FJ480826.1	pigeon	China	2006
EF030952.1	dove	South Africa	2005
EF030951.1	chicken	South Africa	2006
EU240580.1	pigeon	Slovakia	2006
EU240578.1	pigeon	Slovakia	2006
EU240576.1	pigeon	Slovakia	2006

EU240575.1	pigeon	Slovakia	2006
DQ417113.1	pigeon	China	1996
AY471797.1	pigeon	Canada	1991
AY471755.1	pigeon	UK	2002
AY471739.1	pigeon	Denmark	2001
AY471738.1	pigeon	Denmark	2000
AY150160.1	pigeon	Denmark	2001
AY150158.1	pigeon	Sweden	2000
AY150128.1	pigeon	Canada	1992
AY150127.1	pigeon	Canada	1991
AY150124.1	pigeon	Denmark	1990
AY150116.1	pigeon	USA (MN)	1987
AY150110.1	pigeon	Denmark	1987
AY150103.1	pigeon	Hungary	1984
KX247376.1	pigeon	China	2015
KT381602.1	pigeon	China	2013
KT163264.1	pigeon	China	2012
KM374057.1	pigeon	Anhui	2012
KJ600777.1	pigeon	China	2012
JX901318.1	duck	USA (PA)	2002
KC143163.1	pigeon	China	1996
JX901120.1	pigeon	Belgium	2005
HE972209.1	pigeon	Luxembourg	2006
JX486553.1	pigeon	China	2011
JQ775522.1	Eurasian black bird	China	2011
JN942098.1	pigeon	USA (NC)	2000
HM748948.1	chicken	China	2008
GQ281088.1	pigeon	China	2007
GQ281086.1	pigeon	China	2007
GQ281085.1	pigeon	China	2007
FJ766530.1	pigeon	China	2007
FJ766527.1	pigeon	China	2007
FJ766526.1	pigeon	China	2007
GU551934.1	carrier-pigeon	China	2008
EU240582.1	pigeon	Slovakia	2006
AY471794.1	pigeon	Finland	1992
AY471793.1	pigeon	UK	1988
AY471786.1	pigeon	South Africa	1998
AY390291.1	pigeon	China	1996
AY151385.1	chicken	Sudan	1975
AY150156.1	pigeon	Sweden	2000
AY150126.1	pigeon	Sweden	1990
AY150117.1	pigeon	Canada	1988

AY175760.1	pigeon	South Africa	1998
KT381604.1	pigeon	China	2014
KT381603.1	pigeon	China	2013
KT381601.1	pigeon	China	2013
KT381592.1	pigeon	China	2014
KJ607164.1	pigeon	China	2003
JX901341.1	pigeon	USA (PA)	2007
KC013040.1	pigeon	USA (PA)	2007
HG424629.1	pigeon	Nigeria	2013
HG326604.1	pigeon	Nigeria	2009
JX244789.1	pigeon	China	2000
KC143155.1	pigeon	China	1997
JX518532.1	Laughing dove	Kenya	2012
JX486557.1	pigeon	China	2012
JQ290284.1	pigeon	China	2009
JN872173.1	pigeon	USA (NY)	1984
JN967788.1	pigeon	USA (MD)	1985
GQ338311.1	pigeon	China	2005
FJ766528.1	pigeon	China	2005
FJ410147.1	pigeon	USA (MD)	1984
FJ410145.1	pigeon	USA (NY)	1984
FJ480825.1	pigeon	China	2005
EF520716.1	pigeon	USA (NY)	1984
DQ296070.1	pigeon	Turkey	2005
AY471850.1	pigeon	Italy	1984
AY390289.1	pigeon	China	2001
AY150162.1	pigeon	Hungary	2001
AY150122.1	pigeon	Canada	1990
AY150111.1	pigeon	Egypt	1987
AY150107.1	pigeon	Hungary	1986
AF378259.1	pigeon	China	1995
AF001109.1	pigeon	Kuwait	1997
AF001108.1	pigeon	Iraq	1997
KJ544861.1	Human	Netherlands	2003
KM374056.1	pigeon	China	2013
KC143162.1	pigeon	China	1999
KC143158.1	pigeon	China	2003
HE972212.1	pigeon	Luxembourg	2007
JN638237.1	dove	Italy	2010
GQ281087.1	pigeon	China	2007
FJ766531.1	pigeon	China	2007
DQ217716.1	pigeon	China	2003
AY471792.1	pigeon	UK	1991

AY471790.1	pigeon	UK	1989
AY471787.1	swan	Israel	2001
AY390290.1	pigeon	China	1999
AY150136.1	duck	Denmark	1993
AY150113.1	pigeon	Hungary	1987
AY175757.1	pigeon	Ireland	1996
AY175755.1	pigeon	Denmark	1995
AJ880277.1	pigeon	Italy	1982
AB070407.1	pigeon	Japan	1986
AB070394.1	chicken	Japan	1985
AB070390.1	pigeon	Japan	1984
AF001110.1	fowl	Lebanon	1970
KY569334.1	pigeon	Malaysia	2006
KU377532.1	turtle	dove	2008
JN872166.1	dove	Italy	2008
AY471788.1	pigeon	Israel	2001
HG424628.1	pigeon	Nigeria	2013
KR082484.1	pigeon	Egypt	2014
JX901312.1	pigeon	USA (PA)	2001
KC013031.1	pigeon	USA (PA)	2001
HG424627.1	pigeon	Nigeria	2013
KC143167.1	pigeon	China	1997
KC143157.1	pigeon	China	2002
JN872187.1	pigeon	USA (IL)	1993
JN872186.1	pigeon	USA (IN)	1991
JN967786.1	pigeon	USA (NJ)	2000
JF749831.1	pigeon	Uruguay	2008
EU240574.1	pigeon	Slovakia	2006
DQ198273.1	pigeon	China	2002
AY471855.1	pigeon	UK	1984
AY471853.1	rice bran	UK	1984
AY471849.1	pigeon	Belgium	1984
AY471848.1	pigeon	UK	1984
AY471833.1	pigeon	UK	1986
AY471832.1	pigeon	UK	1988
AY390288.1	pigeon	China	1997
AY151384.1	chicken	Sudan	1975
AY150144.1	pigeon	Croatia	1995
AY150138.1	pigeon	Denmark	1993
AY150097.1	pigeon	Denmark	1983
AY150094.1	pigeon	Malta	1982
AY175738.1	falcon	UAE	1996
AB070417.1	pigeon	Japan	1995

KC143161.1	pigeon	China	2005
KC143160.1	pigeon	China	2005
JN942099.1	gannet	USA (PA)	2000
FJ865434.1	pigeon	China	2002
EU296500.1	pigeon	Taiwan	1984
DQ439886.1	pigeon	China	2005
DQ439885.1	pigeon	China	2005
AY471856.1	pigeon	China	1986
AY471836.1	pigeon	UK	1989
AY471789.1	pigeon	Austria	2000
AY390292.1	pigeon	South Africa	1998
AY135749.1	pigeon	Germany	1994
AY151383.1	chicken	Sudan	1975
AY150165.1	turtle dove	Croatia	2002
AY150123.1	Hyacinth macaw	Germany	1990
AY150095.1	pigeon	Germany	1983
AY325798.1	pigeon	China	
AF109885.1	pigeon	UK	1984
AB070416.1	pigeon	Japan	1993
AB070413.1	pigeon	Japan	1991
AB070412.1	pigeon	Japan	1991
AB070409.1	pigeon	Japan	1988
KM588202.1	Eagle	Israel	2014
KC143154.1	pigeon	China	1998
HM627541.1	pigeon	Poland	2010
AY471854.1	pigeon	UK	1986
AY471847.1	pigeon	UK	1984
AY135748.1	Saker	UAE	1990
AY150108.1	pigeon	Germany	1987
AY150101.1	chicken	Germany	1984
AY150098.1	pigeon	Hungary	1983
AY150096.1	pigeon	Germany	1983
AY175768.1	pigeon	UK	1989
AF358785.1	chicken	Singapore	1998
AB070392.1	pigeon	Japan	1984
AB070388.1	chicken	Japan	1981
KU377534.1	pigeon	Italy	2011
KC143150.1	chicken	China	2006
DQ217671.1	chicken	China	2006
AY471827.1	pigeon	UK	1989
AY471812.1	pigeon	UK	Unknown
AY150121.1	dove	Italy	1989
AY150102.1	pigeon	Croatia	1984

AF402131.1	chicken	Bulgaria	1982
AF400615.1	pigeon	China	1997
Y18728.1	pigeon	Russia	1974
AB070391.1	pigeon	Japan	1984
AB070389.1	chicken	Japan	1984
KU885390.1	pigeon	India	2015
KT962979.1	pigeon	Russia	2010
KJ920204.1	pigeon	Russia	2011
KU862297.1	pigeon	Pakistan	2014
KT965727.1	pigeon	Kazakhstan	2014
KT343665.1	pigeon	Iran	2012
JF824047.1	pigeon	Russia	2007
EF543764.1	pigeon	Korea	1999
AY630422.1	pigeon	Korea	1997
AY471835.1	pigeon	UK	1994
AY471829.1	pigeon	Ireland	1990
AY471828.1	pigeon	UK	1990
AY471811.1	pigeon	UK	Unknown
AY471807.1	pigeon	Ireland	1992
AY252120.1	pigeon	Hungary	1990
AY150147.1	pigeon	Italy	1996
AF402116.1	chicken	Bulgaria	1974
KY510683.1	owl	Iceland	2016
KX236101.2	pigeon	Pakistan	2015
KT343676.1	pigeon	Iran	2013
KT343673.1	pigeon	Iran	2013
KT343672.1	pigeon	Iran	2013
KT343670.1	pigeon	Iran	2012
KJ914672.1	pigeon	Ukraine	2011
KC143148.1	chicken	China	2003
JF824030.1	pigeon	Russia	2005
JN967787.1	pigeon	USA (IL)	1987
FJ766529.1	chicken	China	1997
DQ217668.1	pigeon	China	2003
AY630414.1	pigeon	Korea	1989
AY471826.1	pigeon	UK	1989
AY471824.1	pigeon	UK	1986
AY471822.1	pigeon	UK	1988
AY471820.1	pigeon	UK	1988
AY471806.1	chicken	Ireland	1991
AY471748.1	pigeon	UK	1986
AY150125.1	pigeon	Italy	1990
AY175767.1	pigeon	UK	1988

AF234030.1	pigeon	Taiwan	1999
KT343663.1	pigeon	Iran	2012
KF944359.1	pigeon	Israel	2013
KX580988.1	pigeon	Egypt	2015
KX580986.1	pigeon	Egypt	2014
KT343675.1	pigeon	Iran	2013
KT343674.1	pigeon	Iran	2013
KT343671.1	pigeon	Iran	2012
KT343664.1	pigeon	Iran	2012
KT343662.1	pigeon	Iran	2012
AB853928.2	chicken	Japan	1987
KC143147.1	chicken	China	2003
JF824045.1	pigeon	Russia	2008
JF824044.1	pigeon	Russia	2007
AB465606.1	chicken	Japan	1985
DQ296066.1	pigeon	Turkey	2005
DQ217666.1	pigeon	China	2003
AY630413.1	pigeon	Korea	1988
AY471817.1	dove	UK	1996
AY175673.1	pigeon	China	1989
AF458016.1	China	chicken	1986
AF458015.1	China	chicken	1997
AB070397.1	chicken	Japan	1985
AY150120.1	pigeon	Hungary	1989
AY150114.1	pigeon	Hungary	1987
AY471846.1	dove	Italy	2000
AY471837.1	pigeon	Italy	1994
AY471823.1	pigeon	UK	1988
AY471816.1	pigeon	UK	1993
AY150142.1	pigeon	Italy	1994
KU377537.1	pigeon	Italy	2014
KX580976.1	pigeon	Egypt	2013
KT343669.1	pigeon	Iran	2012
KT343668.1	pigeon	Iran	2012
KT343666.1	pigeon	Iran	2012
JF824052.1	pigeon	Russia	2008
AY135742.1	pigeon	Bulgaria	1995
AF458018.1	chicken	China	1997
AF458017.1	chicken	China	1998
AF001131.1	fowl	Sweden	1995
AY150118.1	pigeon	Hungary	1988
KT965728.1	pigeon	Kazakhstan	2014
KC853020.1	crested ibis	China	2010

HQ839733.1	chicken	Sweden	1995
AY630416.1	pigeon	Korea	1990
AY471825.1	pigeon	UK	1990
AY471819.1	pigeon	UK	1990
AY150146.1	pheasant	Italy	1995
AY175769.1	pigeon	UK	1990
AY175758.1	pheasant	Italy	1995
AY175655.1	duck	Tanzania	1995
AF525384.1	pigeon	Germany	1981
AF402117.1	chicken	Bulgaria	1974
AF109878.1	fowl	Denmark	1995
AF001129.1	fowl	Denmark	1995
AF001112.1	fowl	Hungary	1982
KT343678.1	pigeon	Iran	2013
KU377528.1	turtle dove	Italy	2000
KU644586.1	pigeon	Pakistan	2014
KU373026.1	ostrich	China	2006
KU885949.1	pigeon	Pakistan	2014
KT343677.1	pigeon	Iran	2013
JX855038.1	ostrich	China	2006
KC143152.1	chicken	China	1997
EF589137.1	pigeon	China	2007
AY734535.1	pigeon	Argentina	1999
AY471841.1	pigeon	Italy	1996
AY471838.1	pigeon	Italy	1998
AY390296.1	chicken	China	2003
AY150148.1	pigeon	Italy	1996
AY175667.1	Peregrine Falcon	UAE	1992
JX855037.1	crested ibis	China	2010
KC143149.1	chicken	China	2003
DQ217670.1	chicken	China	2003
AY471840.1	pigeon	Italy	1997
AY150145.1	pigeon	Italy	1995
AF458020.1	chicken	China	1991
AF402135.1	chicken	China	1986
AF109879.1	chicken	Sweden	1995
AF001132.1	fowl	Switzerland	Unknown
AY471844.1	pigeon	Austria	1996
KX236100.2	pigeon	Pakistan	2015
KU862298.1	pigeon	Pakistan	2015
KT343667.1	pigeon	Iran	2012
AF458019.1	chicken	China	1997
AF001133.1	fowl	Austria	1996

AF378258.1	pigeon	China	1995
DQ296068.1	pigeon	Turkey	2005
AY471845.1	pigeon	Austria	1996
AF234034.1	pigeon	Taiwan	1999
FJ969394.1	chicken	Egypt	2006
KJ525712.1	chicken	Sudan	2011
AY288997	chicken	Kenya	1990
HM776583	pigeon	China	2008
KJ525675	pigeon	China	2013
KU377533	turtle dove	Italy	2010
KU377535	turtle dove	Italy	2012
KU377536	turtle dove	Italy	2012
KU885390	pigeon	India	2015
KY510683.1	scopowl	Israel	2016
JN872162	rosella	Belgium	2008
JX901120	pigeon	Belgium	2005
KJ736742	pigeon	Germany	1998
AY288995	dove	Italy	2000
GQ429292	pigeon	Ireland	1996
GQ429293	dove	Italy	2000
JN872153	chicken	USA (CA)	1971
EU477188	dove	USA (TX)	2004
EU477189	pigeon	USA (RI)	2000
EU477190	pigeon	USA (TX)	2004
EU477191	ECDO	USA (TX)	2005
EU477192	ECDO	USA (TX)	2005
EU477195	ECDO	USA (TX)	2006
JN872160	pigeon	USA (MN)	2007
JN872167	dove	USA (FL)	2006
JN872170	pigeon	USA (TX)	2010
JN872172	raptor	USA (MN)	2009
JN872174	environment	USA (NJ)	2010
JN872175	pigeon	USA (MN)	2009
JN872176	pheasant	USA (MA)	2005
JN872177	chicken	USA (MA)	2004
JN872178	turkey	USA (LA)	2004
JN872179	pigeon	USA (NV)	2003
JN872190	pigeon	USA (SD)	2007
JN941996	pigeon	USA (PA)	2008
JN941998	pigeon	USA (PA)	2008
JN941999	pigeon	USA (MN)	2008
JN942000	dove	USA (FL)	2008
JN942100	chukar	USA (NJ)	2000

JX901304	pigeon	USA (PA)	2003
JX901305	pigeon	USA (PA)	2003
JX901313	pigeon	USA (PA)	2001
JX901314	dove	USA (PA)	2001
JX901317	pigeon	USA (PA)	2002
JX901319	pigeon	USA (PA)	2005
JX901320	pigeon	USA (PA)	2005
JX901321	chicken	USA (PA)	2005
JX901323	pigeon	USA (PA)	2006
JX901324	pigeon	USA (PA)	2006
JX901325	pigeon	USA (NY)	2006
JX901326	pigeon	USA (NJ)	2006
JX901327	pigeon	USA (PA)	2006
JX901328	pigeon	USA (NJ)	2006
JX901329	pigeon	USA (PA)	2007
JX901330	pigeon	USA (PA)	2007
JX901332	pigeon	USA (MO)	2007
JX901333	pigeon	USA (PA)	2007
JX901334	pigeon	USA (PA)	2007
JX901335	pigeon	USA (PA)	2007
JX901336	pigeon	USA (NC)	2007
JX901337	pigeon	USA (CT)	2007
JX901338	pigeon	USA (OH)	2007
JX901339	pigeon	USA (NJ)	2007
JX901340	pigeon	USA (PA)	2007
JX901342	pigeon	USA (MN)	2007
JX901344	pigeon	USA (ME)	2007
JX901346	pigeon	USA (PA)	2007
JX901347	pigeon	USA (NY)	2007
JX901348	pigeon	USA (PA)	2007
JX901349	pigeon	USA (MD)	2007
JX901350	pigeon	USA (PA)	2007
JX901351	pigeon	USA (NJ)	2007
JX901352	pigeon	USA (PA)	2007
JX901353	pigeon	USA (PA)	2007
JX901354	pigeon	USA (PA)	2007
JX901355	pigeon	USA (PA)	2007
JX901356	pigeon	USA (PA)	2007
JX901357	pigeon	USA (PA)	2007
JX901358	pigeon	USA (PA)	2008
JX901359	pigeon	USA (PA)	2008
JX901360	pigeon	USA (PA)	2008
JX901361	pigeon	USA (PA)	2008

JX901362	pigeon	USA (PA)	2008
JX901363	pigeon	USA (PA)	2008
JX901364	pigeon	USA (PA)	2008
JX901365	pigeon	USA (PA)	2008
JX901366	pigeon	USA (PA)	2008
JX901367	pigeon	USA (PA)	2008
JX901368	pigeon	USA (PA)	2008
JX901369	pigeon	USA (NJ)	2008
JX901372	chicken	USA (PA)	2009
JX901373	pigeon	USA (PA)	2009
JX901374	pigeon	USA (OH)	2009
JX901375	pigeon	USA (PA)	2009
JX901376	pigeon	USA (PA)	2009
JX901377	pigeon	USA (PA)	2009
KP780870	ROPI	USA (MD)	2013
KP780871	ROPI	USA (PA)	2013
KP780872	ROPI	USA (PA)	2013
KP780873	ROPI	USA (PA)	2013
KP780874	ROPI	USA (PA)	2013
KP780875	ROPI	USA (MI)	2013
KP780876	ROPI	USA (MI)	2013
KU059751	pigeon	USA (RI)	2000
KU059752	ECDO	USA (TX)	2004
JN872183	pigeon	USA (MD)	1998
AF109885	domestic fowl	UK	1984
AJ880277	pigeon	Italy	1982
AY734535	pigeon	Argentina	1999
EF520716	pigeon	USA (NY)	1984
FJ410145	pigeon	USA (NY)	1984
FJ410147	pigeon	USA (MD)	1984
FJ865434	pigeon	China	2002
JN872173	pigeon	USA (NY)	2007
JN967787	pigeon	USA (IL)	1987
JN967788	pigeon	USA (MD)	1985
KU377528	turtle	dove	2000
KU377531	pigeon	Italy	2006
AB465606	chicken	Japan	1985
AB853928	chicken	Japan	1987
AF458015	chicken	China	1997
AF458016	chicken	China	1986
AF458017	chicken	China	1998
AF458018	chicken	China	1997
EF589137	pigeon	China	2007

HQ839733	chicken	Sweden	1995
KC853020	crested ibis	China	2010
KU373026	ostrich	China	2006
KY042125	chicken	Bulgaria	1992
AB853926	chicken	Japan	1969
Z12111	chicken	UK	1966
AF458019	chicken	China	1997
AF458020	chicken	China	1991
AF458021	chicken	China	1994
GQ507801	chicken	South Korea	1989
KY042142	quail	Korea	1988
KY042143	chicken	Korea	1993
AY325798	pigeon	China	2003
DQ417113	pigeon	China	1996
FJ480825	pigeon	China	2005
FJ480826	pigeon	China	2006
FJ766528	pigeon	China	2005
GQ338311	pigeon	China	2005
JQ290284	pigeon	China	2009
JX244794	pigeon	China	2008
JX244801	pigeon	China	2008
JX244803	pigeon	China	2008
JX244805	pigeon	China	2008
KJ607163	pigeon	China	2004
KJ607164	pigeon	China	2003
KJ808820	pigeon	China	2012
KT381596	pigeon	China	2013
KX710209.1	pigeon	India	2015
AF358785	pigeon	China	1998
JN872180	water fowl	USA (TX)	2002
JN872182	pigeon	USA (NY)	1998
JN942095	pigeon	USA (OK)	1997
JN942098	pigeon	USA (NC)	2000
JN942099	gannet	USA (PA)	2000
JN967786	pigeon	USA (NJ)	2000
JX901312	pigeon	USA (PA)	2001
JX901318	duck	USA (PA)	2002
JX901341	pigeon	USA (PA)	2007
JN872187	pigeon	USA (IL)	1993
JN872185	pigeon	USA (NY)	1984
JN872186	pigeon	USA (IN)	1991
JN872188	pigeon	USA (MN)	1989
FJ410148	pigeon	USA (TX)	1998

JN872184	pigeon	USA (WY)	1997
JN942022	chicken	USA (TX)	2004
JF824013	pigeon	Russia	2009
JF824032	pigeon	Russia	2005
KJ914671	pigeon	Ukraine	2011
KJ914672	pigeon	Ukraine	2011
KJ920204	pigeon	Russia	2011
KT962979	pigeon	Russia	2010
KT965727	pigeon	Kazakhstan	2014
KT965728	pigeon	Kazakhstan	2014
KT965729	starling	Kazakhstan	2014
KT965730	northern pintail	Kazakhstan	2014
KT965731	Long-legged buzzard	Kazakhstan	2014
KT965732	pigeon	Kazakhstan	2014
KX352835	mallard	Russia	2009
KY042127	pigeon	Ukraine	2013
KY042128	pigeon	Ukraine	2007
KY042129	pigeon	Egypt	2015
KY042130	pigeon	Egypt	2015
KY042131	pigeon	Egypt	2015
KY042132	pigeon	Egypt	2015
KY042133	pigeon	Egypt	2015
KY042134	pigeon	Egypt	2015
KY042136	pigeon	Pakistan	2015
KY042137	pigeon	Pakistan	2015
KY042138	pigeon	Pakistan	2015
KY042139	pigeon	Pakistan	2016
JQ039385	dove	Nigeria	2007
HG326601	pigeon	Nigeria	2007
HG326602	pigeon	Nigeria	2007
HG326603	pigeon	Nigeria	2007
HG326604	pigeon	Nigeria	2009
JX518532	laughing dove	Kenya	2012
KF944359	pigeon	Israel	2013
HG424627	pigeon	Nigeria	2013
JQ039387	pigeon	Nigeria	2008
JQ039391	pigeon	Nigeria	2007
AY734536	pigeon	Argentina	1997
HG424625	pigeon	Nigeria	2013
JN638234	dove	Italy	2011
JN638235	dove	Italy	2011
JN638236	dove	Italy	2010
AY288996	pigeon	Italy	2000

FJ766527	pigeon	China	2007
GQ281085	pigeon	China	2007
GQ281086	pigeon	China	2007
GQ281087	pigeon	China	2007
GQ281088	pigeon	China	2007
GU551934	carrier pigeon	China	2008
HE972209	pigeon	Luxemburg	2006
HE972212	pigeon	Luxemburg	2007
HM063423	white breasted water hen	China	2005
HM063425	wild pigeon	China	2003
HM625835	duck	China	2008
HM748948	chicken	China	2008
JF713701	pigeon	China	2010
JN872166	dove	Italy	2008
JN941995	pigeon	USA (NY)	2008
JN941997	pigeon	USA (FL)	2008
JN986839	pigeon	Ireland	2004
JQ268609	pigeon	China	2011
JX094510	pigeon	China	2012
JX244789	pigeon	China	2000
JX244798	pigeon	China	2008
JX244799	pigeon	China	2008
JX244800	pigeon	China	2008
JX486550	pigeon	China	2011
JX486551	pigeon	China	2010
JX486553	pigeon	China	2011
JX486554	pigeon	China	2011
JX486555	pigeon	China	2011
JX486557	pigeon	China	2012
JX901109	pigeon	Belgium	1998
JX901110	pigeon	Belgium	1998
JX901111	pigeon	Belgium	1998
JX901119	pigeon	Belgium	2005
JX901121	pigeon	Belgium	2007
KJ525672	pigeon	China	2010
KJ525673	pigeon	China	2011
KJ525674	pigeon	China	2013
KJ600777	pigeon	China	2012
KR014815	pigeon	China	2014
KT381595	pigeon	China	2014
KT381606	pigeon	China	2014
KU377529	turtledove	Italy	2002
KU377530	turtledove	Italy	2004

KU377532	turtledove	Italy	2008
KU377534	pigeon	Italy	2011
KU377537	pigeon	Italy	2014
KX247376	pigeon	China	2015
KX761865	pigeon	China	2011
KT163264	pigeon	China	2012
KY042126	chicken	Bulgaria	1982
KM374059	pigeon	China	2013
JQ979176	pigeon	China	2011
JQ993431	pigeon	China	2011
JX486552	pigeon	China	2011
JX901122	pigeon	Belgium	2011
JX901123	pigeon	Belgium	2011
JX901124	pigeon	Belgium	2011
KJ607165	pigeon	China	2013
KJ607166	pigeon	China	2013
KJ782376	pigeon	China	2011
KJ808819	pigeon	China	2013
KM374056	pigeon	China	2013
KM374057	pigeon	China	2012
KM374058	pigeon	China	2012
KM374060	pigeon	China	2011
KM374061	pigeon	China	2012
KP861633	pigeon	China	2012
KT381592	pigeon	China	2014
KT381601	pigeon	China	2013
KT381602	pigeon	China	2013
KT381603	pigeon	China	2013
KT381604	pigeon	China	2014
KU522142	pigeon	Egypt	2014
KU527559	pigeon	China	2015
KU527560	pigeon	China	2015
KT163261	pigeon	China	2012
KT163262	pigeon	China	2013
KT163263	pigeon	China	2013
KX710210.1	pigeon	India	2015
KF828884.1	pigeon	China	2013
KC205475	chicken	Ethiopia	2011
KC205476	chicken	Ethiopia	2011
KC205477	chicken	Ethiopia	2011
KC205478	chicken	Ethiopia	2011
KC205479	chicken	Ethiopia	2011
KJ958913	chicken	Ethiopia	2012

KJ958914	chicken	Ethiopia	2012
KU862297	pigeon	Pakistan	2014
KU862298	pigeon	Pakistan	2015
KU862299	pigeon	Pakistan	2015
KU885949	pigeon	Pakistan	2014
KX236100	pigeon	Pakistan	2015
KX236101	pigeon	Pakistan	2015
KY042135	pigeon	Pakistan	2015
KY042140	pigeon	Pakistan	2016
KY042141	pigeon	Pakistan	2016

*****UK-United Kingdom
*UAE- United Arab Emirates
