# Supplementary material for: Whole-genome sequencing of genotype VI Newcastle disease viruses from formalin-fixed paraffin-embedded tissues from wild pigeons reveals continuous evolution and previously unrecognized genetic diversity in the U.S
Source: Virol J. 2018 Jan 12;15:9. doi: 10.1186/s12985-017-0914-2 (PMC5767055; doi:10.1186/s12985-017-0914-2)
Supplement: Supplementary file 2 — Comparison of next-generation sequencing results to immunohistochemistry results from 48 Eurasian collared-doves and rock pigeons formalin-fixed paraffin-embedded samples collected in the U.S. between 2010 and 2016. (PDF 208 kb) [file 12985_2017_914_MOESM2_ESM.pdf]

Table S4 Comparison of next-generation sequencing results to immunohistochemistry results from 48 Eurasian Collared Doves and Rock Pigeons formalin-fixed paraffin-embedded samples collected in the U.S. between 2010 and 2016.

**Type of tissue**	**Number of samples processed for NGS**	**IHC positive (*n* = 29)**		**IHC negative (*n* = 19)**		**Total NGS +**	**Total NGS -**
						23	25
		NGS +	NGS -	NGS +	NGS -		
Kidney	16	14	1	0	1	14	2
Liver	17	3	2	0	12	3	14
Spleen	15	6	3	0	6	6	9
Total	48	23/29 (79%)	6/29 (21%)	0/19 (0%)	19/19 (100%)	23/48 (48%)	25/48 (52%)
