# Supplementary material for: Whole-genome sequencing of genotype VI Newcastle disease viruses from formalin-fixed paraffin-embedded tissues from wild pigeons reveals continuous evolution and previously unrecognized genetic diversity in the U.S
Source: Virol J. 2018 Jan 12;15:9. doi: 10.1186/s12985-017-0914-2 (PMC5767055; doi:10.1186/s12985-017-0914-2)
Supplement: Supplementary file 3 — The number of raw reads, filtered reads, and mean length of the reads of formalin-fixed paraffin-embedded tissue samples of wild pigeons collected in the U.S. between 2010 and 2016 sequenced by next-generation sequencing (a continuation of Table 1). (PDF 32 kb) [file 12985_2017_914_MOESM3_ESM.pdf]

**Table S5** The number of raw reads, filtered reads, and mean length of the reads of formalin-fixed paraffin-embedded tissue samples of wild pigeons collected in the U.S. between 2010 and 2016 sequenced by next-generation sequencing (a continuation of Table 1).

| SEPRL ID[a] | Raw reads[b] | Filtered reads[c] | Mean fragment length[d] |
|---|---|---|---|
| **1177-1** | **281,595** | **101,867** | **127** |
| **1177-2** | **298,765** | **133,479** | **134** |
| **1177-3** | **273,772** | **97,573** | **124** |
| 1178-2 | 348,758 | 107,760 | 99 |
| 1178-3 | 492,731 | 79,840 | 118 |
| **1179-1** | **468,373** | **142,832** | **134** |
| 1179-2 | 349,992 | 122,874 | 99 |
| 1179-3 | 371,462 | 113,878 | 85 |
| **1180-1** | **323,798** | **82,303** | **146** |
| **1180-2** | **373,021** | **63,076** | **154** |
| **1180-3** | **261,102** | **58,969** | **160** |
| **1181-1** | **789,277** | **358,603** | **226** |
| **1181-2** | **118,942** | **45,840** | **189** |
| **1181-3** | **728,732** | **209,985** | **143** |
| **1182-1** | **282,145** | **74,144** | **201** |
| 1182-2 | 139,459 | 20,759 | 156 |
| **1182-3** | **296,801** | **83,712** | **167** |
| 1183-1 | 102,912 | 18,295 | 135 |
| 1183-3 | 577,717 | 111,807 | 96 |
| **1184-1** | **447,260** | **120,181** | **160** |
| 1184-2 | 675,639 | 196,080 | 99 |
| 1184-3 | 260,637 | 59,975 | 86 |
| **1185-1** | **383,615** | **114,210** | **149** |
| 1185-2 | 441,925 | 120,749 | 96 |
| 1185-3 | 465,822 | 127,284 | 103 |
| 1187-1 | 221,880 | 67,423 | 103 |
| 1187-2 | 535,022 | 169,400 | 107 |
| 1187-3 | 276,923 | 97,278 | 81 |
| **1188-1** | **761,054** | **192,584** | **121** |
| 1188-2 | 662,055 | 146,393 | 120 |
| **1188-3** | **261,977** | **53,863** | **158** |
| **1189-1** | **467,073** | **178,877** | **213** |
| 1189-2 | 70,355 | 20,726 | 93 |

| | | | |
|---|---|---|---|
| 1189-3 | 241,116 | 54,204 | 126 |
| **1191-1** | **474,651** | **226,018** | **213** |
| 1191-2 | 142,706 | 39,172 | 189 |
| **1191-3** | **216,770** | **58,841** | **167** |
| **1192-1** | **274,143** | **37,064** | **162** |
| 1192-2 | 252,619 | 71,948 | 85 |
| 1192-3 | 478,451 | 109,171 | 92 |
| **1193-1** | **131,923** | **62,997** | **197** |
| 1193-2 | 512,056 | 82,248 | 122 |
| **1194-1** | **308,540** | **75,551** | **170** |
| 1194-2 | 357,966 | 49,016 | 135 |
| 1194-3 | 320,925 | 87,458 | 91 |
| **1195-1** | **324,745** | **52,308** | **178** |
| 1195-2 | 152,545 | 46,335 | 176 |
| 1195-3 | 5,988 | 1,976 | 127 |

[a] positive samples are in bold font.

[b] all reads assigned to each sample

[c] the number of paired reads remaining after host and internal control filtering

[d] Average fragment size of the reads in each library
