# Supplementary figures and images for: Whole-genome sequencing of genotype VI Newcastle disease viruses from formalin-fixed paraffin-embedded tissues from wild pigeons reveals continuous evolution and previously unrecognized genetic diversity in the U.S
Source: Virol J. 2018 Jan 12;15:9. doi: 10.1186/s12985-017-0914-2 (PMC5767055; doi:10.1186/s12985-017-0914-2)

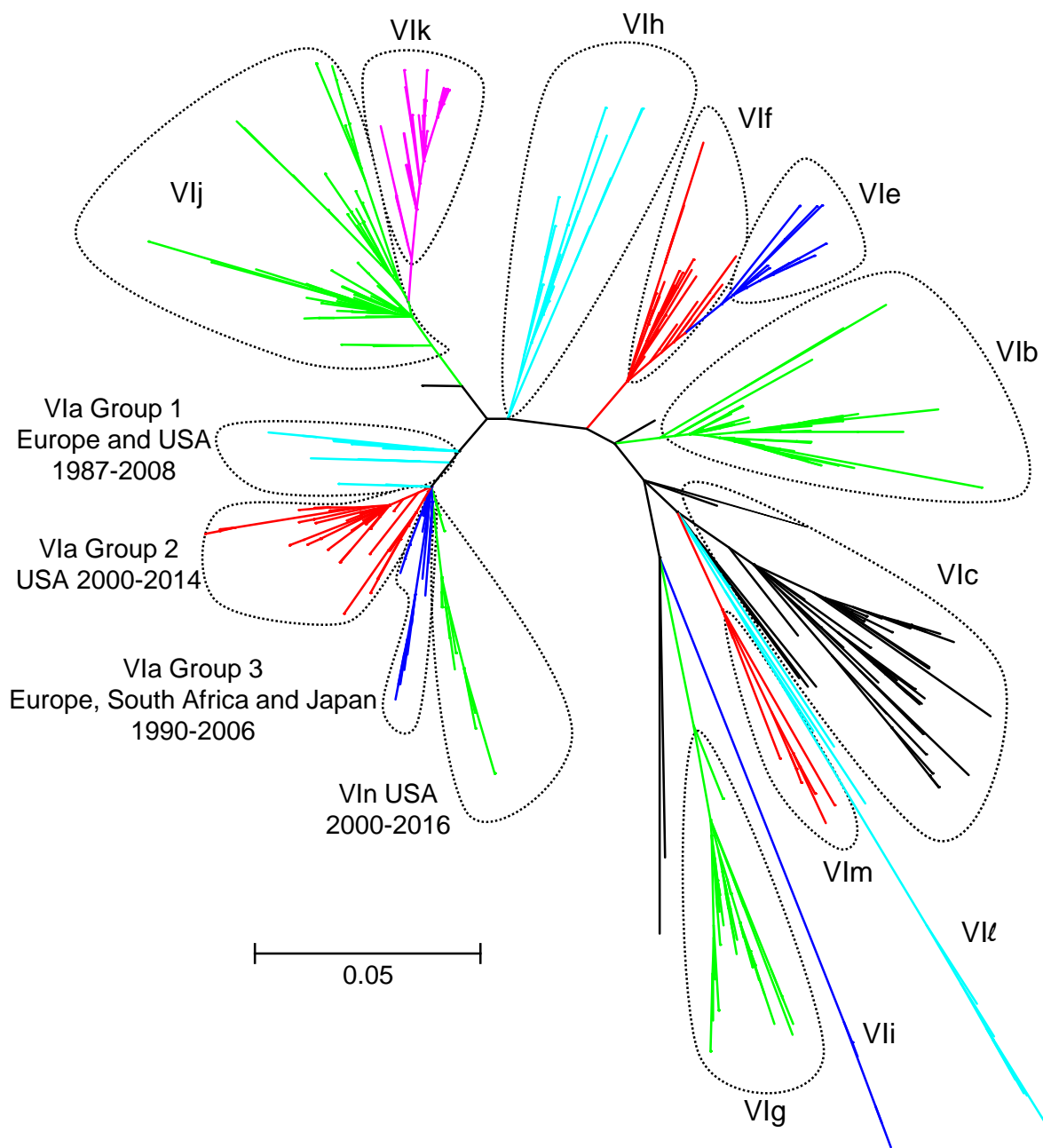

Supplement: Supplementary file 4 — Phylogenetic analysis using the 374-nucleotide partial fusion gene sequences of genotype VI Newcastle disease viruses. The evolutionary history was inferred using the Maximum-likelihood method based on the Kimura-2 parameter model with 1000 bootstrap replicates [42]. The analysis involved 931 genotype VI partial fusion gene sequences (374 nucleotides). Roman numerals are used for the sub-genotype VI designation, and for a clearer view, the taxa names and bootstrap values are not shown in the radiation tree. The VIa and VIn groups are labeled with the country and years of isolate collection. Evolutionary analyses were conducted in MEGA6 [21]. (PDF 283 kb) [file 12985_2017_914_MOESM4_ESM.pdf]
